# Chinese herbal medicines: the modifier of hepatocellular carcinoma targeting Wnt/β-catenin signaling pathway

**DOI:** 10.3389/fphar.2025.1626251

**Published:** 2025-08-25

**Authors:** Yifan Zhang, Hongkun Li, Na Jiang, Qingjing Ru

**Affiliations:** ^1^ The Second School of Clinical Medicine, Zhejiang Chinese Medical University, Hangzhou, China; ^2^ Hangzhou Institute for Advanced Study, University of Chinese Academy of Sciences, Hangzhou, China; ^3^ The Second Affiliated Hospital of Zhejiang Chinese Medical University, TCM Hepatology Department, Hangzhou, China

**Keywords:** Hepatocellular carcinoma, Wnt/β-catenin signaling pathway, traditional Chinese medicine, immunity, chemotherapy drugs

## Abstract

Hepatocellular carcinoma (HCC) is a prevalent malignant neoplasm of the digestive system, including 80% of primary liver malignancies. The Wnt/β-catenin signaling pathway plays a key role in immune response and tumer resistance. A growing number of studies have shown that the Wnt/β-catenin signaling pathway is involved in the pathogenesis of HCC. The Wnt/β-catenin pathway affects HCC in a variety of ways, including by influencing gene mutations, regulating dendritic cells, T-cells, and tumor cells, and influencing tumer resistance. Presently, conventional chemotherapy exhibits several drawbacks, including side effects, restrictions, and the emergence of tumer resistance. Traditional Chinese medicine (TCM) are characterized by multiple actions, multiple targets, few side effects, and improved immunity, and their combination with common clinical therapies can prolong patient survival and reduce postoperative recurrence rates, providing a new idea of combination therapy for the alleviation and improvement of HCC. This review focuses on TCM as an adjunct to surgery, targeted therapy, interventional therapy to improve the HCC microenvironment, reverse tumer resistance, and reduce treatment side effects by modulating the Wnt signaling pathway. It should be clear that TCM should not replace the first-line treatment plan of modern medicine, and its core value is to improve the comprehensive efficacy and quality of life of patients. This research examines the role of the Wnt/β-catenin signalling system in developing HCC and describes how TCM and plant active metabolites, crude extracts of single botanical drugs and Chinese herbal formulations affect the progression of HCC by modulating different targets of the Wnt/β-catenin signalling pathway or by modulating other pathways related to the Wnt/β-catenin signalling pathway. This review is intended to provide new ideas and options for the prevention and treatment of HCC.

## Graphical Abstract

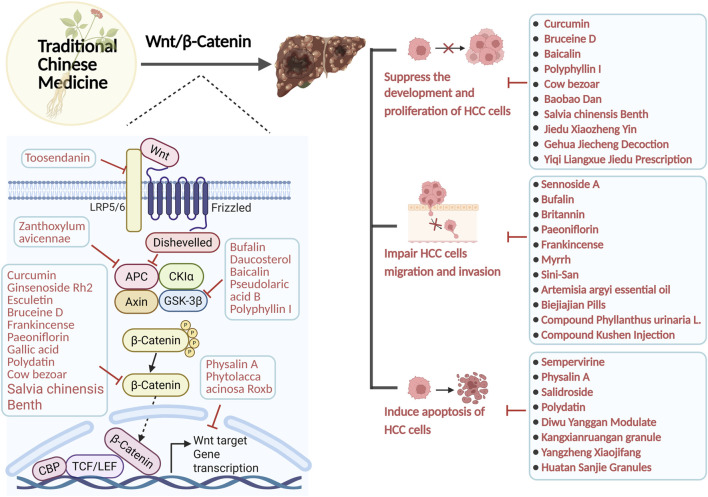

## Highlights

• The role of the Wnt/β-catenin signaling pathway in the pathogenesis of Hepatocellular carcinoma (HCC) and its critical role in the immune response and tumer resistance were investigated.
• Explored the mechanism of Traditional Chinese medicine (TCM) regulation of HCC treatment through different targets of the Wnt/β-catenin signaling pathway.
• Elucidated the complex effects of TCM and plant active metabolites, crude extracts of single botanical drugs, and Chinese herbal formulas on HCC treatment through the Wnt/β-catenin signaling pathway, emphasizing their dual ability to regulate molecular mechanisms and enhance therapeutic potential when combined with conventional therapies.

## 1 Introduction

**FIGURE 1:**
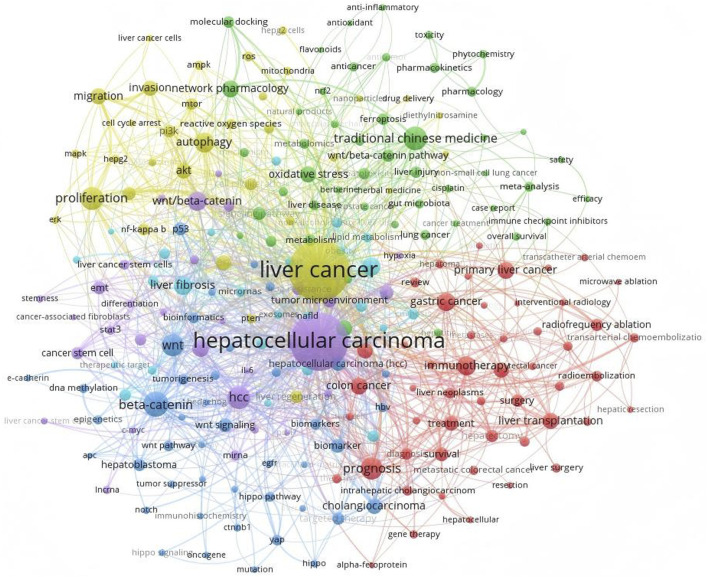
Utilizing VOSviewer to examine the keywords in HCC research reveals that TCM and the Wnt/β-catenin signalling pathway hold significant prominence and represent current research focal points.

The most recent cancer census indicates that liver cancer ranks as the sixth most prevalent malignant tumour globally and is the third primary cause of cancer-related mortality (Bray et al., 2024). Primary liver cancer includes HCC, cholangiocarcinoma (CCA), and mixed liver cancer (Mejia and Pasko, 2020; Yang et al., 2024). Due to the clinical rarity of CCA and mixed liver cancer and the lack of research, this article focuses on HCC. Hepatocellular carcinoma is a prevalent malignant neoplasm of the digestive system, including 80% of primary liver malignancies (Ranković and Hauptman, 2023). It originates from chronic liver damage caused by a combination of factors (Xue et al., 2024). The factors encompass internal and exterior elements, including genetic predisposition, viral or non-viral influences, and the cellular microenvironment (Llovet et al., 2021). Genomic research indicated that gain-of-function mutations in Catenin Beta 1 (CTNNB1) (Cai et al., 2024), which encodes the β-catenin protein, and loss-of-function mutations in Axis inhibition protein 1 (Axin1) were identified in 35% of human HCC samples (Xu et al., 2022). The Wnt/β-catenin pathway collaborates with various signalling pathways, significantly influencing the development and progression of HCC, impacting cell proliferation, differentiation, apoptosis, migration, invasion, and additional processes (Xu et al., 2022; Carson and Nejak-Bowen, 2025). Consequently, targeting the Wnt/β-catenin signalling system has emerged as a novel method for the treatment of HCC (Figure 1 presents current hot keywords for the research of HCC). Currently, several pharmacological agents targeting the Wnt signalling pathway have been identified and investigated, including the small molecule inhibitors pkf115-854 and CGP049090, which inhibit the interaction between β-catenin and T cell-specific transcription factor (Tcf)4 (Yan et al., 2017; Zhao et al., 2023). This anti-Wnt2 monoclonal antibody obstructs Wnt binding to the frizzled (FZD) receptor (Liu H. Y. et al., 2024; Chuan et al., 2024), and the anti-FZD monoclonal antibody OMP-18R5 (Xie W. et al., 2021; Zhang et al., 2023), among others. Nonetheless, the therapeutic efficacy of these medications has not been clinically validated (Yamada et al., 2021; Kim et al., 2024). Consequently, conventional approaches, including surgical procedures like liver resection or transplantation, interventional therapy, local ablation therapy, chemotherapy, and targeted immunotherapy, remain the favoured treatment modalities in clinical practice (Rizzo et al., 2022; Deng et al., 2023). Conventional chemotherapy and radiotherapy have resulted in adverse responses. Post-radiotherapy, patients may exhibit symptoms including pruritus, nausea, emesis, oral mucositis, constipation, diarrhea, and gastrointestinal hemorrhage (Wang et al., 2024). Furthermore, HCC exhibits significant resistance to numerous chemotherapeutic agents (Bukowski et al., 2020). Despite prior findings indicating that the targeted therapies sorafenib and regorafenib exhibit significant efficacy in advanced HCC, prolonged administration of these agents frequently results in tumer resistance, hence influencing later disease progression (Fan et al., 2022). Due to the intricate pathophysiology of HCC, targeted therapies remain underdeveloped and have restricted applicability, albeit continuous research efforts (Xue et al., 2024).

In this context, TCM, a significant aspect of complementary medicine, offers a novel approach to treating HCC (Wu Q. et al., 2022; Li H. et al., 2024). Several *in vivo* and *ex vivo* experiments have investigated the mechanism of TCM in HCC inhibition: a variety of TCM can inhibit the proliferation and invasion of HCC cells and induce apoptosis of HCC cells by acting on the upstream and downstream targets of the Wnt/β-catenin signalling pathway, or by directly regulating the metabolism of β-catenin (Xu et al., 2024; Liang et al., 2024). In addition, TCM is now widely used as an complementary therapy in the clinical treatment of cancer, which can improve the survival rate as well as the quality of life of the patients, and improve the adverse effects. For example, a retrospective cohort study suggests that an integrative treatment approach combining the use of TCM and chemotherapy may be able to improve the survival of patients with intermediate- and advanced-stage HCC, especially for patients with good physical status and Child-Pugh class A (Sun et al., 2018; Liu et al., 2019). Clinical studies have also observed that Jian Pi Li Gan Decoction improves survival as well as prognosis in patients with non-resected HCC after RFA treatment (Tang et al., 2018). As well as the administration of the Fufang Banmao capsule may also improve the survival of patients with advanced HCC and Vp3-4 PVTT receiving supportive treatment time, especially in patients in the high-risk group (score ≥84) (Liu et al., 2020; Hu Y. et al., 2024). Furthermore, the combination of TCM with transcatheter arterial chemoembolization (TACE) and targeted agents demonstrated a 50% increase in the objective response rate (ORR) for advanced HCC compared to TCM monotherapy, substantiating the adjunctive therapeutic value of TCM (Sun et al., 2018). Notably, this review focuses on TCM as an adjunct to surgery, targeted therapy, and interventional therapy. It ameliorates the HCC microenvironment, reverses tumer resistance, and mitigates treatment-related adverse effects by modulating the Wnt/β-catenin signalling pathway. It is imperative to clarify that TCM can be used as an adjunct therapy, not as the primary or sole treatment for HCC. Its core value lies in enhancing overall therapeutic efficacy and patient quality of life.

This review examined publications published in the previous 5 years in the PubMed, Web of Science, Google Scholar, CNKI database and WanFang database, utilizing the keywords ‘Chinese herbal medicine’, ‘TCM’, ‘natural products’, ‘TCM active metabolites’, ‘Chinese herbal formulas’, ‘HCC’,‘Liver cancer’ and ‘Wnt/β-catenin pathway’. Over 300 research papers and review articles were identified and examined. However, existing studies are fragmented and unsystematic. This article offers a detailed examination of the distinctive function of Wnt/β-catenin in HCC. We revealed that the Wnt/β-catenin signaling pathway plays a key role in immune response and tumer resistance. Simultaneously, we have performed a comprehensive analysis of the various impacts of TCM and plant active metabolites, crude extracts of single botanical drugs, and Chinese herbal formulas on HCC through the modulation of several targets within the Wnt/β-catenin signalling pathway. This article is intended to provide new ideas and options for the prevention and treatment of HCC.

## 2 Wnt/β-catenin signalling pathway

**FIGURE 2:**
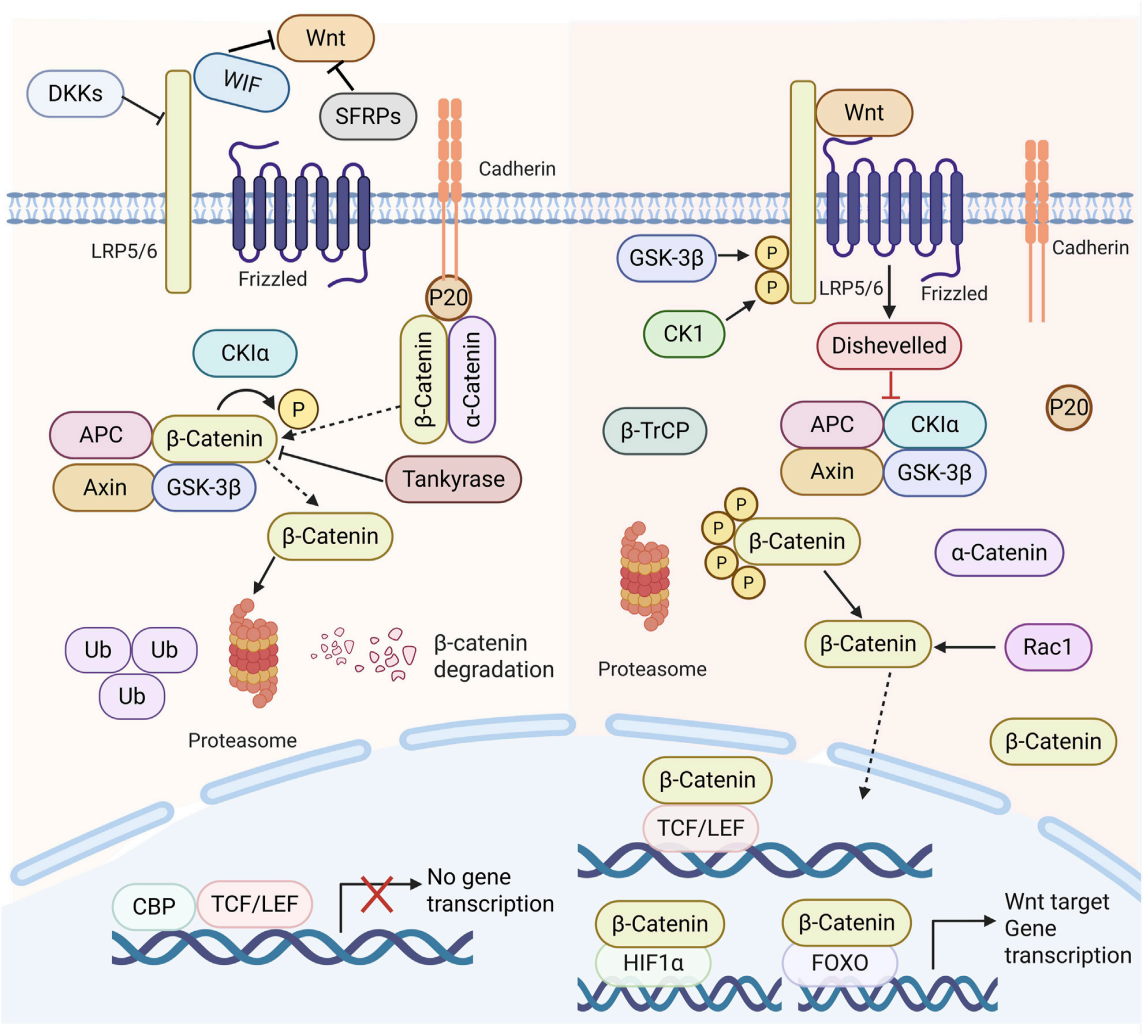
Diagram of the Wnt signalling system in mammals. In the absence of Wnt ligands, β-catenin is phosphorylated and then destroyed in the proteasome complex, resulting in diminished cytoplasmic levels of β-catenin, which precludes the transcription of Wnt target genes. In the presence of Wnt ligands, they interact with frizzled receptors FZD and low-density lipoprotein receptor-related proteins 5/6, leading to the disassembly of the proteasome complex (APC, Axin, GSK3β, Casein kinase 1α (CK 1α). This process allows β-catenin to evade degradation, accumulating in the cytoplasm and subsequent translocation to the nucleus, which associates with TCF/lymphoid enhancer factor (LEF) family members. This interaction promotes the expression of Wnt target genes and regulates cell proliferation, invasion, migration, and the cell cycle.

The Wnt signalling pathway is typically categorized into a canonical pathway (β-catenin-dependent) (This is reflected in Figure 2) and a non-canonical pathway (β-catenin-independent) (Krishnamurthy and Kurzrock, 2018). The non-canonical Wnt signalling pathway primarily facilitates cytoskeletal remodelling and cellular motility (Shi, 2024). In contrast, the canonical pathway (Wnt/β-catenin) is implicated in several critical biological processes, such as embryonic development, adult stem cell maintenance, and regulating proliferation and angiogenesis (Leung and Lee, 2022). The Wnt/β-catenin signalling pathway is prevalent in cells and works to regulate inflammatory responses, prevent cardiovascular disease, and have anti-tumour properties, among others. This route is crucial in the progression of numerous illnesses (Wang S. et al., 2021; Wang Q. et al., 2021). For instance, it influences the development and progression of viral hepatitis and fatty liver disease. Viral hepatitis, particularly HBV infection, increases the risk of HCC development by approximately 100- to 200-fold (Singal et al., 2020). Beyond maintaining HBV transcription and replication, HBx plays a significant role in activating the Wnt/β-catenin signaling pathway within infected hepatocytes. HBx modulates multiple components of the Wnt/β-catenin pathway at both extracellular and intracellular levels. Extracellularly, HBx markedly reduces the expression of Wnt antagonists SFRP1 and SFRP5 by recruiting DNA methyltransferases 1 and 3A to their gene promoters for epigenetic silencing (Xie et al., 2014). Intracellularly, HBx disrupts the β-catenin destruction complex function by competitively binding APC or inhibiting GSK3 activity through Src kinase activation (Hsieh et al., 2011), and by inducing the cell cycle-related kinase-mediated androgen receptor signaling pathway (Yu et al., 2014).

The HCV core protein enhances Wnt/β-catenin signaling activation within the nucleus. This is achieved by increasing the expression levels of Wnt ligands, FZD, and LRP5/6 receptors (Fukutomi et al., 2005), while simultaneously downregulating the transcription of Wnt antagonists SFRP2 and DKK1 (Umer et al., 2014). Furthermore, the HCV core protein promotes hypermethylation of the CDH1 gene promoter region (Ripoli et al., 2011), leading to reduced E-cadherin expression. Consequently, less β-catenin is sequestered at the cell membrane by the β-catenin/E-cadherin complex, resulting in elevated levels of free cytoplasmic β-catenin and enhanced Wnt/β-catenin pathway activation. HCV also augments Wnt/β-catenin signaling independently. HCV infection upregulates microRNA-155 (miR-155) expression, which directly suppresses APC expression (Zhang et al., 2012). Additionally, HCV infection increases signaling through the epidermal growth factor receptor (EGFR) and fibroblast growth factor (FGF) pathways. Both pathways contribute to β-catenin stabilization via tyrosine phosphorylation at residue Tyr654 and inactivation of GSK3β, stimulated through the PI3K/Akt and Ras/Raf/MEK/ERK cascades, thereby releasing β-catenin from the β-catenin/E-cadherin complex (Igloi et al., 2015).

NAFLD is also a major risk factor for the development and progression of HCC (Bhala et al., 2011). The Wnt/β-catenin signaling pathway is dynamically and finely regulated throughout the progression from NASH to NAFLD and subsequently to HCC. Aberrant lipogenesis, a core event in NAFLD, requires the transcription factor peroxisome proliferator-activated receptor gamma (PPARγ). However, the Wnt/β-catenin signaling pathway suppresses PPARγ mRNA expression (Ross et al., 2000). Thus, its inactivation is necessary for NAFLD development, as evidenced by dyslipidemia and fatty liver disease in mice carrying non-conservative inactivating mutations in the Wnt coreceptor LRP6, and the rescue of NAFLD by Wnt ligand Wnt3a (Go et al., 2014).

Increased intrahepatocellular lipid burden, oxidative stress, and lipid peroxidation induce hepatic inflammation and fibrosis, which can escalate to NASH. During this process, overexpressed aortic carboxypeptidase-like protein (ACLP) restores Wnt/β-catenin signaling by specifically binding to FZD8 and LRP6 to form a ternary complex, thereby promoting extracellular signal transduction (Go et al., 2014). Wnt ligand levels are further augmented by complementary secretion from infiltrating macrophages. Furthermore, epigenetic modifications of involved components contribute to Wnt/β-catenin pathway activation, including methylation of Wnt antagonists, histone deacetylation at the AXIN2 promoter, and downregulation of microRNAs that negatively regulate Wnt/β-catenin signaling (Wang S. et al., 2015).

## 3 Role of the Wnt/β-catenin pathway in the pathogenesis of HCC

### 3.1 Impact on the occurrence of HCC

The Wnt/β-catenin signalling pathway is integral to HCC pathophysiology (Rana et al., 2019). The Wnt/β-catenin signalling pathway activates in 20%–35% of HCC cases (Jemal et al., 2010), primarily due to mutations in critical genes like CTNNB1, Axin, and APC (Xu et al., 2022). Mutations in CTNNB1, which encodes β-catenin, can impede β-catenin phosphorylation and subsequent degradation, stimulating the Wnt/β-catenin signalling pathway, and enhancing cell proliferation and motility (Deldar Abad Paskeh et al., 2021). Loss-of-function mutations in Axin impair Axin within the β-catenin degradation complex, resulting in dysregulation of the Wnt/β-catenin signalling pathway and disturbance of cellular homeostasis (Liang et al., 2023). A functional loss-of-function mutation in APC results in the excessive accumulation of β-catenin (Khalaf et al., 2018), causing sustained aberrant stimulation of the Wnt/β-catenin signalling pathway, which disrupts processes such as proliferation, apoptosis, and cell motility. Moreover, the etiological factors of HCC, including the HBV virus, can induce mutations in genes associated with the transduction of the Wnt/β-catenin signalling pathway (Yeh et al., 2023). Research indicates that the Wnt/β-catenin signalling system influences cell proliferation, invasion, metastasis, and self-renewal in HCC cells (Gajos-Michniewicz and Czyz, 2024). Sun et al. discovered that Klotho overexpression adversely modulates the Wnt/β-catenin signalling pathway, diminishes endogenous β-catenin levels, and obstructs its nuclear translocation, thereby retarding cell cycle progression (Sun et al., 2015). Metabolic reprogramming of glucosylceramide can stimulate the Wnt/β-catenin signalling pathway, resulting in decreased expression of GBA1, which subsequently facilitates epithelial-mesenchymal transition (EMT) and augments the metastatic potential of HCC (Qiu et al., 2022). Furthermore, Wang et al. discovered that the inhibition of the Wnt/β-catenin signalling pathway resulted in a marked decrease in the surface markers of cancer stem cells, suggesting that the blockade of Wnt/β-catenin impedes the development of spheroid-forming HCC stem cells (Wang et al., 2016).

### 3.2 Effects on the immune system

Clinical studies have identified the Wnt/β-catenin pathway as one of the major pathways (Ruiz de Galarreta et al., 2019) affecting the efficiency of current immune checkpoint therapies, and activation of the Wnt/β-catenin pathway has been associated with lower rates of disease control, progression-free survival, and overall survival in HCC patients treated with immune checkpoint inhibitors (ICIs) (Harding et al., 2019). In addition to its direct involvement in carcinogenesis, the Wnt/β-catenin signaling pathway is also involved in tumor immune escape (Fu et al., 2015; Spranger and Gajewski, 2015). Cancer cell antigens are usually recognized by dendritic cells (DCs), which activate B cells to produce antibodies DCs also induce differentiation of primed T cells into cytotoxic effector T cells, which are recruited to the tumor site to kill cancer cells (Spranger et al., 2015; Spitzer et al., 2017; Wang B. et al., 2018). In contrast, upregulation of Wnt/β-catenin signaling helps tumors evade immune surveillance and render chemotherapy and immunotherapy ineffective and/or resistant, thereby increasing the likelihood of recurrence (Galluzzi et al., 2019).

The final step in the tumor-immune cycle is the recognition and killing of tumor cells by effector T cells, and the most common strategies used by tumor cells to evade immune attack are the expression of the negative regulatory molecule programmed cell death 1(PD-1)/L1 and the generation of mutant tumor antigens through immunoediting (Blank et al., 2004). The expression of PD-L1 has been shown to be regulated by MYC, a downstream target in the Wnt/β-catenin signaling pathway. Therefore, blocking the Wnt/β-catenin signaling pathway in cancer cells will inhibit the expression of CD47 and PD-L1, and thus enhance the anti-tumor immune response (Wong et al., 2015).

### 3.3 Impact on tumor resistance

**FIGURE 3:**
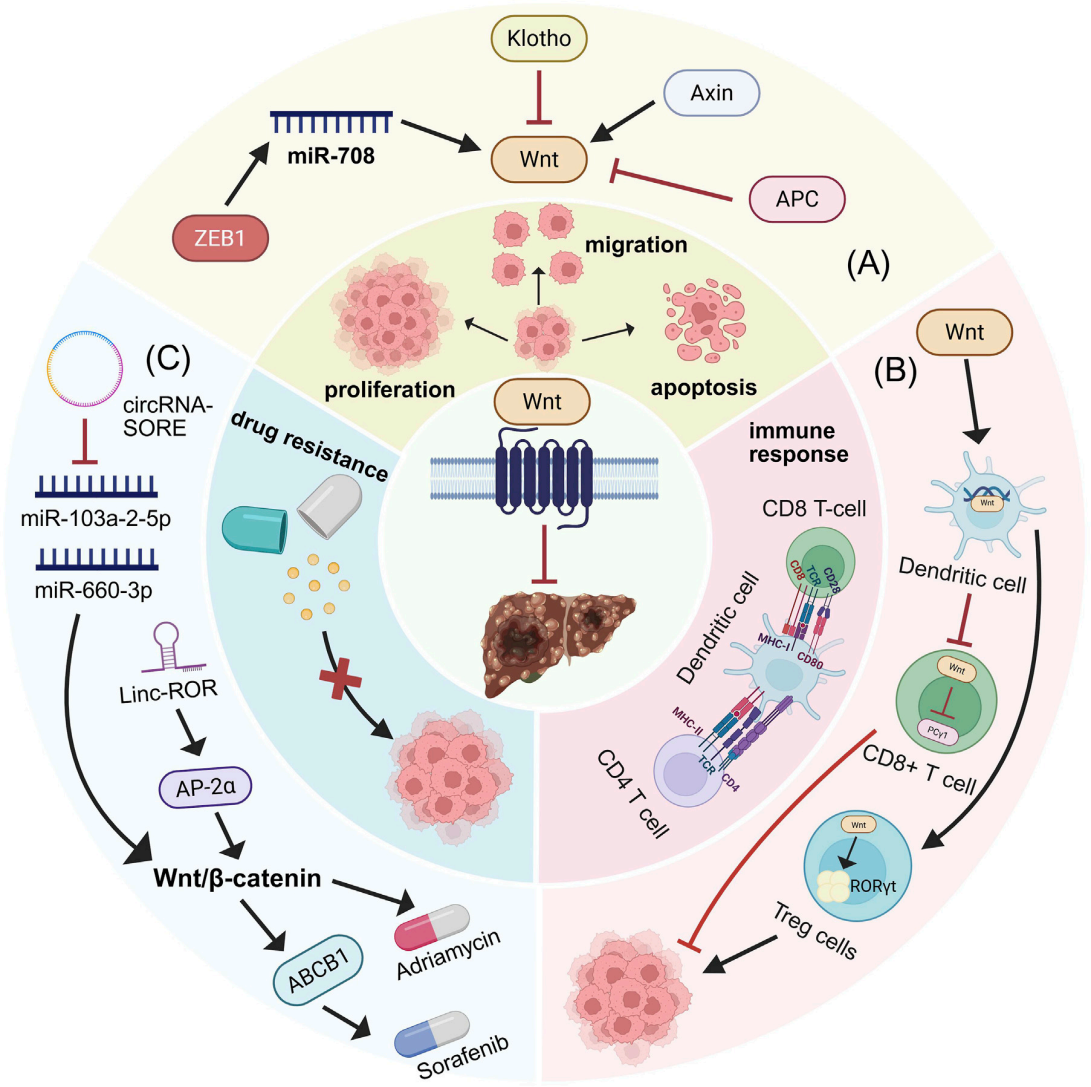
Wnt/β-catenin signaling pathway regulation of HCC biological behaviors. **(A)** Biomolecules targeting the Wnt/β-catenin signaling pathway modulate HCC proliferation, invasion, and apoptosis. **(B)** Role of Wnt/β-catenin signaling pathway in immunity. Wnt ligands within the tumor microenvironment impair DC function, thereby suppressing the priming and activation of CD8^+^ cytotoxic T cells and preventing their anti-tumor activity. β-catenin in CD8^+^ T cells reduces phospholipase C-gamma 1 activity, which is essential for T cell activation. Furthermore, Wnt/β-catenin activation in CD4^+^ regulatory T cells (Tregs) induces RORγt expression, promoting immune evasion. **(C)** Contribution of Wnt/β-catenin signaling pathway to tumer resistance.

The Wnt/β-catenin signaling pathway has been shown to play a crucial role in regulating tumor resistance (Zhou et al., 2022). In HCC, activation of the Wnt/β-catenin pathway impairs dendritic cell recruitment and reduces T-cell activity, thereby promoting immune evasion of HCC cells and inducing resistance to ICIs such as PD-1 (Ruiz de Galarreta et al., 2019). A recent study analyzing tumor samples from 41 sorafenib-treated patients found that Wnt/β-catenin activation correlated with sorafenib resistance, with elevated nuclear β-catenin levels predicting a poor response (He et al., 2025). Mechanistic investigations revealed that in sorafenib-resistant cells, increased levels of circRNA-SORE bind to miR-103a-2–5p and miR-660–3p, competitively activating the Wnt/β-catenin pathway to induce sorafenib resistance (Xu et al., 2020). Furthermore, LincROR interacts with AP-2α, activating the Wnt/β-catenin pathway and increasing HCC resistance to doxorubicin. Wnt3a-induced activation of the Wnt/β-catenin pathway also confers resistance to regorafenib in HCC(Shi C. J. et al., 2023). In summary, multiple mechanisms converge to promote immune evasion and contribute to increased HCC tumer resistance, thereby diminishing treatment efficacy, primarily through modulation of Wnt/β-catenin pathway activity. Identifying the mechanisms underlying immune evasion and developing inhibitors to disrupt this process are crucial for improving HCC therapy (as illustrated in Figure 3).

Research demonstrates that Bruceine D enhances the efficacy of sorafenib in a time- and dose-dependent manner. Mechanistic studies revealed that Bruceine D interferes with the formation of the β-catenin/TCF-4 complex and significantly reduces the expression levels of Wnt/β-catenin target genes (e.g., LEF1, Survivin, Axin-2, c-Myc), thereby augmenting the antitumor effects of sorafenib both *in vitro* and *in vivo* (Cheng et al., 2017). Further investigation identified that Bruceine D targets the β-catenin-binding protein ICAT, blocking its interaction with β-catenin, promoting β-catenin degradation, and inhibiting HIF-1α-mediated glucose metabolism, consequently overcoming chemoresistance (Huang et al., 2021).

Additionally, studies found that Curcumin reverses sorafenib resistance by inhibiting β-catenin nuclear translocation and downregulating EMT markers (e.g., N-cadherin, Vimentin) and lncRNA expression (Zhu et al., 2022). Matrine effectively downregulates the Wnt-1/β-catenin pathway, reverses EMT, alleviates intrahepatic inflammation, counteracts immune exhaustion, reduces chemotherapeutic drug efflux, and reverses HCC multidrug resistance (Shi J. et al., 2023). Jiedu Recipe was found to inhibit the Wnt/β-catenin pathway under hypoxic conditions, downregulate stemness transcription factors (NANOG, OCT4, SOX2) and Cripto-1 protein expression, block EMT and CSC properties, and reduce tumor recurrence and tumer resistance (Guo et al., 2023). Jianpi Huayu Formula enhances sensitivity to 5-FU by targeting circular RNA circβ-catenin, disrupting its interaction with the Wnt pathway, and upregulating E-cadherin while downregulating Vimentin/N-cadherin. Furthermore, Jianpi Huayu Formula downregulates Piezo1 expression and inhibits the PI3K/AKT/mTOR pathway, sensitizing HCC to doxorubicin (Fang et al., 2025).

## 4 The conventional perspective and therapeutic approach of TCM for HCC

In conjunction with pertinent records from ancient TCM texts of prior dynasties, including those found in the “Treatise on the Origin and Syndrome of Diseases” (诸病源候论): “Consequently, the inferior section of the ribs is distended and painful, and the body exhibits a yellow hue (故胁下满痛而身发黄)”, as stated in “The Scripture of Difficulties: Fifty-six Difficulties” (难经·五十六难): The liver’s buildup is referred to as fat Qi, persisting for years. It can be categorized as an illness under “Liver Qi stagnation,” “tympanites,” “jaundice,” and “swelling and pain beneath the ribs” in TCM. Hepatocellular cancer is attributed to the accumulation of phlegm-toxins, damp-heat, and blood stasis in the liver, resulting from deficiencies in the liver’s vital energy, external pathogenic influences, irregular dietary habits, and emotional distress (Li Y. et al., 2022). In the initial phases of HCC, the primary pathogenic mechanism is the liver’s impaired ability to regulate Qi flow and the consequent Qi stagnation. This phase may exhibit no clinical manifestations and is challenging to identify. In the intermediate phases of liver disease, it is usual for both the liver and spleen to be simultaneously damaged, resulting in “wood stagnation and earth deficiency” (木郁土虚). Symptoms including stomach distension, diminished appetite, and diarrhea frequently mark this phase (Wu Y. et al., 2022). Clinical practice has revealed that patients with HCC frequently have digestive symptoms as the initial manifestation (Jia and Wang, 2020). In the advanced stages, cancer metastasizes, the positive energy diminishes, and the negative energy intensifies; qi and blood (TCM) become severely depleted, concurrently impacting the liver, spleen, and kidneys. The TCM approach to HCC prioritizes syndrome identification and therapy, judicious drug selection and application, and a mix of reinforcement and elimination while addressing both symptoms and underlying causes. It significantly alleviates patient pain, prevents tumour recurrence, and extends lifespan (Lu Q. et al., 2024).

Presently, clinically employed therapies exhibit drawbacks, including adverse effects, constraints, and the emergence of tumer resistance (Craig et al., 2020). TCM employs a holistic methodology and a diagnostic and therapeutic strategy grounded in syndrome differentiation (Wen et al., 2024). It emphasizes differentiating various syndromes and, based on the relevant treatment principles and methods for each syndrome, incorporates factors such as the patient’s age, gender, and constitution to create a personalized prescription, termed “one person, one prescription.” (Wang et al., 2020; Wu et al., 2021). Traditional Chinese medicine seeks to equilibrate yin and yang, modulate qi and blood circulation (vital energy), preserve the body’s health, and facilitate disease treatment and health maintenance (Tian et al., 2024). As complementary therapy, it has been shown to be effective in combination with conventional treatments in mitigating the side effects of chemotherapy, improving postoperative recovery, and reducing the risk of recurrence and metastasis (Lai et al., 2023). For example, clinical trials have found that the combination of Fuzheng Jiedu Xiaoji formulation and TACE can significantly prolong the overall survival and progression-free survival of patients with HCC and reduce mortality (Yang X. et al., 2021). The Chinese herbal medicine formulas PHY906 or YIV-906 has shown good therapeutic effects and reduced the toxicity of capecitabine in phase I/II clinical trials of combination therapy. Similarly, Changou et al. also found that PHY906 combined with capecitabine had a good therapeutic effect, with a disease stabilization rate of 46.2% and a median overall survival of 6 months (Changou et al., 2021). Tsai et al. also found that patients using the Chinese botanical drugs had a 37% lower risk of developing HCC than those who did not use Chinese medicine (Tsai et al., 2017). There is a large body of epidemiological evidence that diabetes is associated with the development of several cancers, especially HCC (Niwa et al., 2015; Qiao et al., 2016). Lu et al. found in a retrospective cohort study that the adjunctive use of TCM can effectively reduce the incidence of HCC in diabetic patients (adjusted hazard ratio [aHR] 0.59). Hepatitis B cirrhosis with hyperalphafetoproteinemia is an intermediate stage of liver cirrhosis that can progress to HCC (Taura et al., 2012). Clinical trials have found that the Erzhu jiedu recipe can significantly inhibit the serum AFP and AFP-L3 levels of patients with hepatitis B cirrhosis and hyperalphafetoproteinemia, and has good safety, which is expected to inhibit the occurrence of HCC (Chen et al., 2021). Thus, TCM is increasingly recognised as an important part of comprehensive liver cancer treatment (Tan et al., 2023).

## 5 Traditional Chinese medicine and plant active metabolites influence the progression of HCC by regulating multiple targets of the Wnt/β-catenin signalling pathway

A large number of *in vivo* studies have shown that certain plant active metabolites and TCM can influence the progression of HCC by targeting multiple targets of the Wnt/β-catenin signaling pathway. It is important to note that the metabolites discussed herein are not exclusive to TCM and are also present in food and plants. However, their specific profile, concentration, or synergistic interactions within this TCM formulation may contribute to its observed effects. For example, some active metabolites, including toosendanin (Yang et al., 2021b), bufalin (Gai et al., 2016; Zhang X. et al., 2024), daucosterol (Zeng et al., 2017), baicalin (Sun et al., 2023), Pseudolaric Acid B (Zhang H. et al., 2019), and polyphyllin I (Liao et al., 2022), along with crude extracts of single botanical drugs such as Zanthoxylum Avicenna (Wu et al., 2017), as well as Chinese herbal formulas like Babao Dan (Xie X. et al., 2021) and Diwu Yanggan Modulates (Zhao B. B. et al., 2019), can target upstream elements of the Wnt/β-catenin pathway, specifically LRP6, GSK-3β, and APC. This interaction diminishes β-catenin production, consequently inhibiting HCC’s proliferation, and metastasis. Metabolites from TCM, including curcumin (Shao et al., 2020), ginsenoside Rh2 (Xu et al., 2024), Bruceine D (Ha et al., 2021), Paeoniflorin (Yang L. et al., 2020), gallic acid (Xie et al., 2024), polydatin (Jiao et al., 2018), can directly interact with the β-catenin protein, leading to a reduction in β-catenin expression and the inhibition of HCC proliferation, migration, and invasion. Metabolites from plants and TCM, including Icaritin (Zhao H. et al., 2015), Isoliquiritigenin (Huang et al., 2020), Physalin A (Shang et al., 2024), along with crude extracts of single botanical drugs such as Phytolacca acinosa Roxb (Liu J. et al., 2024), Cow bezoar (Zeng et al., 2017), and Chinese herbal formulas like Sinikangai fang (Guo et al., 2022) and Yanggan Huayu granule (Shen et al., 2022), target downstream genes such as Cyclin D1, c-myc, and B-cell lymphoma-2 (Bcl-2) within the Wnt/β-catenin signaling pathway, thereby inhibiting the cell cycle of HCC cells and disrupting their proliferation. These results indicate that the mechanism of action of TCM and plant active metabolites in the treatment of HCC is intricately linked to Wnt/β-catenin. Consequently, suppressing the hyperactivation of the Wnt/β-catenin signalling system will constitute a novel research direction to treating HCC with TCM.

## 6 Traditional Chinese medicine and plant active metabolites modulate Wnt/β-catenin signaling pathway to influence the progression of HCC

Numerous *in vivo* and clinical studies indicate that active metabolites of plant and TCM have various effects in treating HCC by inhibiting the proliferation of HCC cells and their migration and invasion, while also inducing apoptosis through the Wnt/β-catenin signalling pathway (Yu et al., 2022). It is important to note that the metabolites discussed herein (e.g., Bruceine D, Polyphyllin I) are not exclusive to TCM and also found in plants and other traditional medicines, etc. But these metabolites have been identified as key components in the studied TCM.

### 6.1 Suppress the growth of HCC

Curcumin is among the most extensively researched metabolites derived from the rhizome of Curcuma longa. A phase II clinical trial investigated the immunotherapeutic effect of a combination of curcumin, piperine, and taurine (CPT) on HCC. The results showed that after CPT administration, the levels of miRNA-21, IL-6, VEGF-α, LDH, and alpha-fetoprotein (AFP) were significantly reduced compared to baseline levels. Therefore, CPT may have a potential immunostimulatory effect in patients with HCC (Kotb et al., 2023). (Clinical trial: 26 HCC patients, p. o., 5 g/d, 3 months). However, this study utilized a self-controlled design (pre-vs post-treatment comparison) without including untreated control or active comparator groups. Furthermore, it did not evaluate the immunomodulatory effects of CPT across different HCC grades or stages, warranting further investigation. *In vivo* experiments have also shown that curcumin can inhibit the recruitment of Axin to the cell membrane in a concentration-dependent manner, disrupt the complex that stabilizes β-catenin, and prevent β-catenin accumulation in cells, thereby obstructing its interaction with LEF/TCF. This interruption of Wnt signalling subsequently reduces the expression of β-catenin target genes such as c-myc, Vascular Endothelial Growth Factor (VEGF), and cyclin D1, thereby inhibiting the proliferation of BEL-7402 and QGY-7703 cells and inducing apoptosis in these cells (*In vivo*, Mice,i.m., 25 days, PBC (negative control)). Curcumin possesses excellent physiological functions; however, due to its poor water solubility and photosensitivity, it degrades under strong light exposure. Therefore, identifying stable and bioactive curcumin formulations is a crucial step toward advancing its clinical applications.

Brucea javanica (L.) Merr. (Simaroubaceae) has been approved in China for the treatment of gastrointestinal tumours, hepatocellular carcinoma, and lung cancer (Fan et al., 2020). Bruceine D (BD) is an active metabolite isolated and extracted from Brucea javanica (L.) Merr. (Simaroubaceae), and has been found to exhibit cytotoxic and antiproliferative effects against pancreatic cancer, breast cancer, lung cancer, leukaemia, osteosarcoma, and HCC (Sin et al., 2020). Furthermore, Bruceine D is a Wnt inhibitor (Huang et al., 2021) that modulates the expression of Wnt target genes Lef1, Survivin, Axin2, and c-Myc while also inhibiting Wnt/TCF4-mediated translational activity both *in vivo* and *in vitro*, hence inhibiting the proliferation of Huh7 and Hep3B cells (*In vivo*, Mice, i. v., 0.75 mg/kg–1.5 mg/kg, 10 days, Saline (negative control)). Although Bruceine D exhibited inhibitory effects even at high concentrations (10 and 15 μmol/L), this experiment lacked an active comparator. Further studies incorporating positive control groups are required to validate the scientific robustness of the conclusions.

GSK-3β is a downstream signalling target of ROCK1 and an essential element of the β-catenin degradation complex (Sun et al., 2022). Baicalin, a flavonoid derived from the dried root of Scutellaria baicalensis Georgi (Ke et al., 2019), inhibits the proliferation of Hep3B, MHCC-97H, and LO2 cells by downregulating ROCK1 expression, upregulating GSK-3β, and suppressing β-catenin expression, while also inducing cell cycle arrest at the G0/G1 phase and apoptosis (*In vivo*, Mice, gavage, 80 mg/kg, 20 days). However, the *in vivo* component of this study lacked an active comparator group. Furthermore, while the results demonstrate that Baicalin modulates total ROCK1 expression levels, it remains to be determined whether this regulation occurs at the transcriptional level or through post-translational protein modifications. Further research is warranted to elucidate the precise molecular mechanisms underlying Baicalin’s anti-HCC effects. In addition, a meta-analysis showed that herbal preparations with baicalin as the main metabolite, combined with TACE or primary treatment, can significantly improve the objective tumour response rate (risk ratio (RR) = 1.57, 95% confidence interval (CI): [1.30, 1.90], p < 0.00001). Baicalin can slow tumour growth *in vivo*. Based on experimental and clinical evidence, Scutellaria baicalensis Georgi and its active metabolites have the potential to treat HCCin terms of efficacy and safety (Huang et al., 2020). However, baicalin has poor water solubility and low bioavailability, which limits its clinical application. New preparation methods such as nanoemulsions and self-micro emulsifiable drug delivery systems (SMEDDS) can be developed to improve its bioavailability and enhance its efficacy in clinical applications for cancer treatment.

The PI3K/AKT and Wnt/β-catenin pathways share numerous interconnected proteins, including GSK3β, FZD, DVL, Deptor, and eIF4E, which can modulate one another and influence the assembly and disassembly of β-catenin protein phosphorylation and degradation complexes, Wnt receptor expression, and β-catenin protein nuclear translocation (Prajapati and Doshi, 2023). p-AKT is an upstream molecule that phosphorylates GSK-3β at the Ser9 position, resulting in GSK-3β inactivation and subsequent buildup of β-catenin protein (Samant et al., 2023). Polyphyllin I (PPI), a steroidal saponin derived from Rhizoma Paridis, clinical studies have shown that patients with HCC who received treatment with Rhizoma Paridis exhibited a reduction in vasculogenic mimicry in tumour tissue. This study demonstrates the potential of TCM for the treatment of HCC and may contribute to the future development of new anti-HCC drugs from TCM (Xiao et al., 2018). (Clinical trial: 89 HCC patients, p. o., 60 mg/kg/day, 10 days, untreated control group) *In vivo* experiments have also found that both PPI and sorafenib suppressed tumor growth in mice, while the PPI-Sora combination group exhibited significantly enhanced suppression of tumor growth. Mechanistic investigation revealed that PPI can activate AKT/GSK-3β-mediated β-catenin ubiquitin-proteasome degradation and significantly suppresses growth of tumor, (*In vivo*, Mice, i. p., 1 mg/kg, 4 weeks, Saline (negative control), Sora (positive control)). However, this study employed only a single concentration of PPI. Further investigation is warranted to determine whether varying concentrations of PPI exert beneficial effects against HCC and to establish its minimal effective concentration. In addition, as a natural saponin metabolite with a specific structure, Polyphyllin I should possess specific binding characteristics and catalytic affinity for targets based on cellular and molecular chemistry, potentially exhibiting different effects on various cell types (Tian et al., 2020). Therefore, subsequent studies could employ multiple cell lines or primary cells for systematic pathway analysis to enhance the scientific rigor of the conclusions (Other examples are shown in Table 1).

**TABLE 1:** Traditional Chinese medicine and plants active metabolites target the Wnt/β-catenin signalling pathway to suppresses the growth of HCC.

Metabolite name	Source	Chemical formula	Extraction method	Cell	Laboratory animal	Injection method	Dose	Experimental period	Control method	Function	Mechanism of action	References
Curcumin	Curcuma longa L	C_21_H_20_O_6_	—	BEL-7402and QGY-7703 cells	—	—	—	—	Negaive control	Inhibits cell proliferation and metastasis, and induces apoptosis	β-catenin and downstream target genes ↓	Xu et al. (2013)
				SMMC7721 and Huh-7cells						Inhibits cell proliferation and apoptosis	lincROR/β-cateninand downstream target genes ↓	Shao et al. (2020)
				Hep3B, HepG2, SMMC-7721, HUH7, SK-Hep1, MHCC-LM3, MHCC-97H and NCTC-1496 cells	Mice	i.m	—	25d	UNC119 (positive control) and PBC (negative control)	Suppresses the growth, migration and invasion of HCC	β-catenin, UNC119, CCND1, CCNE1 and TGF-β/EMT↓	Zhao et al. (2019b)
Mangiferin	Mangifera indica L	C_19_H_18_O_11_	—	MHCC97L and HLF cells	Mice	p.o	50 mg/kg/2 d	5weeks	Solvent control (negative control)	Suppresses growth and invasion of HCC *in vitro*	LEF1, LEF1 transactivates gene and WT1 ↓	Yu et al. (2022), Tan et al. (2018)
Icaritin	Epimedium sagittatum (Siebold and Zucc.) Maxim	C_21_H_22_O_7_	—	PLC/PRF/5, Huh7 and Hep-12 cells	Mice	i.g	70 mg/kg	40d	Cisplatin, sorafenib (positive control)	Suppresses growth of HCC and induce apoptosis	p-Stat3, Jak2, p-ERK1/2, Stat3 downstream genes Mcl-1, CyclinD1 and IL-6 receptor↓	Zheng et al. (2021), Li et al. (2021a)
Baicalin	Scutellaria baicalensis Georgi	C_21_H_18_O_11_	—	Hep3B, MHCC-97H and LO2 cells	Mice	Gavage	80 mg/kg	20 d	LV-NC (positive control)	Suppresses growth, differentiation, invasion and metastasis of HCC, while inducing cell cycle arrest at the G0/G1 phase and apoptosis	ROCK1/GSK-3β/β-catenin, Bcl-2 and C-myc ↓	Sun et al. (2022), Ke et al. (2019)
Bruceine D	Brucea javanica (L.) Merr	C_20_H_26_O_9_	—	Huh7 and Hep3B cells	Mice	i.v	0.75–1.5 mg/kg	10 d	Saline (negative control)	Suppresses the growth and apoptosis of HCC	Wnt/β-catenin target genes, β-catenin/TCF4, Jagged1 and Axin2 ↓	Cheng et al. (2017), Huang et al. (2021)
Ophiopogonin-B	Ophiopogon japonicus (Thunb.) Ker Gawl	C_39_H_62_O_12_	—	HHL-5, MHC and 97-H cells	Mice	p.o	15 mg/kg-75 mg/kg	21 d	Saline (negative control)	Suppresses growth, migration, invasion and angiogenesis, and promotes apoptosis of HCC	PTP1B, PI3K/AKT ↓,caspase3/Bax↑, AMPK↑	Yuan et al. (2022)
Columbamine	Coptis chinensis Franch	C_20_H_20_NO_4_	—	HCC SMMC7721, HepG2and Hep3B cells	Mice	—	5–20 mg/kg	21 d	Saline (negative control)	Suppresses growth and migration, induces apoptosis of HCC	BAD↑,PCNA, BCL2, PI3K/AKT, ERK1/2, MAPK↓	Tang et al. (2023)
Polyphyllin I	Paris polyphylla Sm	C_44_H_70_O_16_	—	HepG2, Huh-7 and LCSCs cells	Mice	i.p	1 mg/kg	31d	Saline (negative control), Sora (positive control)	Suppresses growth of HCC	AKT/GSK-3β↑, EpCAM, CD13 and β-catenin↓	Shan et al. (2024), Lin et al. (2019)

### 6.2 Impair HCC cellular migration and invasion

Research indicates that numerous beneficial metabolites in TCM can impede the migration and invasion of HCC cells by modulating the Wnt/β-catenin signalling pathway, enhancing therapeutic efficacy against HCC (Liang et al., 2024) (As shown in Table 2).

**TABLE 2:** Traditional Chinese medicine and plant active metabolites target the Wnt/β-catenin signalling pathway to impair the invasion and migration of HCC.

Metabolite name	Source	Chemical formula	Extraction method	Cell	Laboratory animal	Injection method	Dose	Experimental period	Control method	Function	Mechanism of action	References
Bufalin	*Bufo gargarizans*	C_24_H_34_O_4_	—	HepG2.2.15, PLC5, Huh7, HepG2 and HLF cells	Mice	i.v	20 μg/kg	5 weeks	Vehicle (negative control)	Suppresses growth, invasion and migration	GSK-3β Ser9 phosphorylation, β-catenin translocation, cyclin D1, MMP-7 and COX-2↓	Gai et al. (2016), Zhang et al. (2024a), Sheng et al. (2021)
Toosendanin	Melia azedarach L	C_30_H_38_O_11_	—	SMMC-7721 and MHCC-97L cells	Mice	i.v.t	—	4 weeks	Normal mice (blank control)	Suppresses growth of HCC	JAK2/Stat3, FZD8, p-LRP6, DVL2, β-catenin, c-myc and MMPs↓	Yang et al. (2025), Yang et al. (2021c)
Sennoside A	Rheum officinale Baill	C_42_H_38_O_20_	—	HepG2 and SMMC-7721 cells	Mice	i.p	10 mg/kg/d	14d	Saline (negative control)	Suppresses growth and invasion	Wnt, TNF, VEGF and NF-κB↓	Le et al. (2020)
Salidroside	Rhodiola rosea L	C_14_H_20_O_7_	—	HepG2, MHCC97-H (97H), Hs 578Bst and GES-1 cellS	Mice	i.p	Sal(80 mg/kg),CQ (5 mg/kg)	4 weeks	PBS (negative control)	Inhibits invasion and metastasis of HCC cells and induces autophagy	PI3K/AKT/mTOR↓	Jiang et al. (2023a), Lu et al. (2019)
Rosmarinic Acid	Salvia rosmarinus Spenn	C_18_H_16_O_8_	—	HepG2 cells	Mice	i.g	75–300 mg/kg	10d	Cyclophosphamide (positive control)	Inhibits the invasion and metastasis of HCC cells	PI3K/Akt/NF-κB↓ Wnt/β-catenin↓	Cao et al. (2019a)
Germacranolide	Wrightia arborea (Dennst.) Mabb	C_17_H_22_O_4_	—	Hep3B and HepG2 cells	Mice	i.p	5–10 mg/kg/2d	14d	Oxaliplatin (positive control)	Inhibits the migration, EMT and drug resistance of HCC cells	PI3K/AKT, RAS/RAF/MEK/ERK↓	Li et al. (2024c)
Isoliquiritigenin	Glycyrrhizae Radix (Licorice)	C_15_H_12_O_4_	—	Hep3B cells	Mice	i.p	50 mg/kg/1d	21d	Excipient (negative control)	Inhibits migration by G1/S cell cycle transition	PI3K/AKT, cyclinD1↓	Song et al. (2020), Wang et al. (2019)
Britannin	Inula japonica Thunb	C_19_H_26_O_7_	—	PLC/PRF/5, HCC-LM3, Huh-7 and H22 cells	Mice	i.g	7–28 mg/kg/d	21d	Gan-Fu-Le (positive control); normal saline (negative control)	Suppresses growth, migration, and invasion of HCC while promoting their apoptosis	p-GSK-3β, β-catenin, N-Cadherin↓, E-cadherin↑	Lu et al. (2024a)
Paeoniflorin	Paeonia lactiflora root	C_23_H_28_O_11_	—	HepG2 and SMMC-7721 cells	Mice	i.g	25 mg/kg	12d	normal saline (negative control), Sorafenib (positive control)	Suppresses growth and migration of HCC	NF-κB, 5-HT1D, Wnt/β-catenin↓	Zhou et al. (2021), Li et al. (2022b)

Rhubarb is a TCM botanical drug with a long history and wide recognition. It is widely used to treat diseases such as constipation, jaundice, gastrointestinal bleeding, and ulcers. Currently, many Chinese herbal formulas containing rhubarb have been used in the clinical treatment of liver diseases, inflammation, and cancer (Li et al., 2009). Sennoside A is the main anthraquinone active metabolite in Rheum officinale Baill. Its potential therapeutic effects in the treatment of various diseases such as obesity, insulin resistance, hepatic steatosis and HCC (Wei et al., 2020; Le et al., 2020). In addition, the study found that SA inhibited HepG2 cell proliferation and intrahepatic metastasis in a had no significant inhibitory effect on SMMC-7721 cell proliferation and metastasis, indicating that the effect of SA on HCC may be cell-type specific (*In vivo*, Mice, i. p., 10 mg/kg/d, 14 days, Saline (negative control)). However, the *in vivo* arm of this study lacked an active comparator. The inclusion of positive control groups is warranted to further corroborate the therapeutic efficacy of Sennoside A against HCC. In addition, network pharmacology experiments have found that SA mainly exerts its effect by inhibiting the activation of the Wnt, TNF, VEGF, and NF-κB signaling pathways (Le et al., 2020). However, the gastrointestinal side effects associated with long-term and high-dose use of SA remain controversial (Le et al., 2021). It is necessary to further identify relevant targets and conduct clinical trials on the safety, efficacy, and pharmacokinetics of SA to validate the results observed *in vitro* and *in vivo*.

Inula japonica Thunb was approved in the 2020 Edition of the Pharmacopoeia of the People’s Republic of China for the treatment of cough, wheezing, expectoration, and vomiting. Britannin is its important active metabolite (Bailly, 2021) and has been proven to have anti-inflammatory effects, reducing myocardial and cerebral ischemia-reperfusion injury, and anticancer effects (Bailly, 2021). Studies have found that Britannin regulates ROS activation of AMPK to induce cell apoptosis (Lu et al., 2022), and can also inhibit HCC by suppressing p65 protein expression and reducing the Bcl-2/Bax ratio (Li et al., 2020). Additionally, recent studies have found that britannin upregulates the expression of GSK-3β and E-cadherin in a time-dependent manner, while downregulating the expression of p-GSK-3β, β-catenin, and N-Cadherin, thereby reducing the occurrence of EMT and significantly inhibiting HCC metastasis. *In vivo* experiments showed that after Gan-Fu-Le and britannin treatment, the number of tumour cells in liver tissue decreased, accompanied by minimal necrosis and inflammatory infiltration cells. Britannin elicited a more pronounced therapeutic effect than Gan-Fu-Le, suggesting its potential to suppresses HCC growth and metastasis. (*In vivo*, Mice, i. g., 7–28 mg/kg/d, 21 days, Gan-Fu-Le (positive control), normal saline (negative control)). However, the tissue specificity of britannin requires further investigation (Lu Q. et al., 2024). Additional animal models could be established to elucidate the mechanism by which britannin inhibits HCC metastasis.

The overexpression of HT1D markedly elevated the Wnt/β-catenin pathway-associated proteins, such as β-catenin, survivin, C-myc, and cyclin D1 in HCC cells (Fatima et al., 2016). Paeoniflorin (PF) is a monoterpenoid glycoside and the primary metabolite of the root of Paeonia lactiflora Pall. Recent studies have shown that paeoniflorin exhibits a wide range of activities, including liver protection, alleviation of bile stasis, reduction of liver fibrosis, prevention of non-alcoholic fatty liver disease, and inhibition of hepatocellular carcinoma, involving multiple pathways. (He and Dai, 2011). Preclinical studies have shown that PF exhibits antitumour activity against HCC and regulates immune function and suppresses tumour growth through multiple pathways (Lu et al., 2014; Zhou et al., 2021). Research revealed that the combination of paeoniflorin and sorafenib exerted synergistic antitumor effects compared to sorafenib monotherapy. This combination significantly increased CD4^+^ and CD8^+^ T cell infiltration within tumor tissue, markedly enhanced the cytotoxic activity of tumor-specific cytotoxic T lymphocytes (CTLs), and reversed both IL-2 depletion and elevated PD-L1 expression induced by sorafenib intervention. Furthermore, the combined regimen reduced peripheral blood IFN-γ levels and suppressed tumor tissue expression of NF-κB and PD-L1 (*In vivo*, Mice, i. g., 25 mg/kg/d, 12 days; Sorafenib (positive control), Normal saline (negative control)) (Li J. et al., 2024). Subsequent clinical studies in HCC patients are warranted to validate and expand the clinical value of paeoniflorin. In addition, PF has been shown to inhibit the proliferation and migration of HepG2 and SMMC-7721 cells by downregulating 5-HT1D, hence obstructing the expression of the Wnt/β-catenin pathway (Zhou et al., 2021).

GSK-3β phosphorylates the N-terminal β-catenin at Ser33, Ser37, and Thr41 residues, hence designating β-catenin for proteasomal destruction (Li C. et al., 2021; Reabroi et al., 2018). Consequently, numerous active metabolites of TCM can impede the Wnt/β-catenin signalling pathway by enhancing the breakdown of β-catenin through the inhibition of GSK-3β phosphorylation or by augmenting its activity, thereby demonstrating an anti-HCC action. Bufalin is a steroid derivative and the primary active metabolite of the TCM Chansu, extracted from the skin and parotid glands of toads. Research indicates that Bufalin exhibits antitumour activity against various cancers, including HCC (Li H. et al., 2018). In addition, it was found that bufalin induces apoptosis in human HCC cells by targeting JNK activation and Fas-mediated pathways (Qi et al., 2011). Zhuo Yu et al. found that bufalin inhibits PLC5 HCC cell proliferation, transformation, and cell cycle progression, but not LO2 cells, which is related to CCRK-mediated β-catenin/TCF signalling (Yu et al., 2018), Molecular docking experiments remain warranted to further validate the underlying molecular mechanisms. In a mouse model of hepatitis B virus-associated HCC, bufalin downregulates the expression of androgen receptors and cell cycle-related kinases in the β-catenin/TCF signalling pathway, thereby directly killing HCC cells (Yu Z. et al., 2020). In addition, Bufalin can block the phosphorylation of GSK-3β at Ser9, diminish the expression of β-catenin, cyclin D1, metalloproteinase-7, and cyclooxygenase on the cell membrane, regulate EMT, and impede the invasion and migration of BEL-7402 cells (Gai et al., 2016; Zhang X. et al., 2024) (*In vivo*, Mice, i.v., 20 μg/kg, 5 weeks, Vehicle (negative control)). Subsequent clinical studies in HCC patients are warranted to validate and expand the clinical value of Bufalin.

### 6.3 Induce apoptosis of HCC cells

Research in the development of HCC therapeutics has identified the active metabolites of TCM and plant as a potentially significant resource. A growing body of data indicates that the induction of apoptosis may be a primary molecular mechanism by which these natural metabolites combat HCC (Jiang et al., 2023b) (As shown in Table 3).

**TABLE 3:** Traditional Chinese medicine and plant active metabolites target the Wnt/β-catenin signalling pathway to induce apoptosis in HCC.

Metabolite name	Source	Chemical formula	Extraction method	Cell	Laboratory animal	Injection method	Dose	Experimental period	Control method	Function	Mechanism of action	References
Gallic acid	Rosa gallica L	C_7_H_6_O_5_	—	HepG2 cells	Mice	i.g.,i.p	80 mg/kg	4 weeks	normal saline (negative control)	Induces ferroptosis in cells	Wnt/β-catenin/SLC7A11/GPX4↓	Xie et al. (2024), Shi et al. (2021)
Polydatin	Polygonum aviculare L	C_20_H_22_O_8_	—	HepG2 and SMMC-7721 cells	Mice	i.p	25–100 mg/kg	20d	PBS buffer (negative control)	Induces HCC cell apoptosis	β-catenin, c-myc, cyclinD1↓	Jiao et al. (2018)
Physalin A	Physalis alkekengi var. Franchetii (Solanaceae)	C_28_H_30_O_10_	—	HepG2 cells	Mice	i.p	40 mg/kg	28d	PBS buffer (negative control)	Impairs the viability of HepG2 cells and promotes apoptosis and autophagy	PI3K/Akt, Wnt/β-catenin↓	Shang et al. (2024)
Sempervirine	Gelsemium elegans Benth. (*G. elegans*)	C_19_H_17_N_2_	Continuous reflux extraction method	HepG2 and Huh7 cells	Mice	i.p	1 mg/kg	2 weeks	Sorafenib (positive control)	Suppresses growth and induces apoptosis	p53↑, Wnt/β-catenin↓	Yue et al. (2021)
Celastrol	Tripterygium wilfordii Hook.f	C_29_H_38_O_4_	—	HepG2 and Hepa3B cell lines	Mice	i.p	2 mg/kg, twice a week	16d	saline (negative control)	Suppresses growth and induce apoptosis	CXCR4, PI3K/Akt, Wnt/β-catenin↓	Kun-Ming et al. (2020)
Sinomenine	Sinomenium acutum Rehd. et Wils. (Fam. Menispermaceae)	C_19_H_24_O_4_	—	SK-Hep-1 cells	Mice	i.p	75–150 mg/kg/d	20d	Saline (negative control)	Induces apoptosis	PI3K/AKT, Wnt/β-catenin↓	Luo et al. (2022)
Curcumin	Curcuma longa L	C_21_H_20_O_6_	—	HepG2 cells	Mice	i.p	200 mg/kg/d	1 month	Equal volume of solvent (negative control)	Suppresses growth and induces apoptosis	Wnt/β-catenin, GPC3, catenin, c-myc and cyclin D1↓	Hu et al. (2019)
Oroxin B	Oroxylum indicum (L.) Kurz	C_27_H_30_O_15_	—	Hep G2cells	Mice	i.g	4–24 mg/kg/d	16 weeks	Normal mice (blank control), cyclophosphamide (positive control)	Induce apoptosis of HCC	PI3K/Akt, Wnt/β-catenin↓	Li et al. (2019)

Gelsemium elegans (*G. elegans*) Benth is a TCM often used to treat neuropathic pain and cancer (Wang Y. et al., 2015). Research on its active metabolites has found that all species are rich in alkaloids, especially indole alkaloids (Jin et al., 2014). Among them, the structurally representative alkaloids sempervirine, gelsemine, humantene, and koumine have anti-tumor, analgesic, anti-inflammatory and immunomodulatory pharmacological activities (Jin et al., 2021). *In vivo* studies demonstrated that both combination therapy with sorafenib (10 mg/kg) and sempervirine, as well as high-dose sorafenib monotherapy (30 mg/kg), significantly induced tumor cell apoptosis. The combination regimen exerted superior efficacy to high-dose sorafenib alone. Further studies have found that sempervirine can significantly inhibit the nuclear aggregation level of β-catenin and inhibit the transcriptional level of the Wnt pathway, which in turn downregulates its downstream genes cyclin D1, cyclin B1 and CDK2, blocks the G1 phase of the cell cycle and ultimately induces apoptosis in HCC cells (*In vivo*, Mice, i. p., 1 mg/kg, 2 weeks, Sorafenib (positive control)) (Yue et al., 2021). However, this study exclusively evaluated sempervirine at 1 mg/kg without conducting dose-ranging experiments. Subsequent investigations should delineate the impact of varying sempervirine concentrations on HCC to determine whether therapeutic efficacy exhibits concentration dependence.

Physalis alkekengi var. Franchetii is used to treat various diseases such as sore throat, fever, and urinary tract problems (Liang et al., 2022). Physalin A (PA) is a bioactive sterol found in it. Previous studies have shown that PA has various pharmacological activities such as analgesic, anti-inflammatory, antifungal, and chondroprotective activities (Shin et al., 2019). More importantly, PA has been shown to have antitumor properties in various cancers, including non-small cell lung cancer and human fibrosarcoma (Zhu et al., 2016). In addition, studies have found that PA can reduce HepG2 cell viability in a dose-dependent manner by inhibiting the PI3K/Akt signaling pathway, thereby indirectly affecting the Wnt/β-catenin signaling pathway, and promoting apoptosis and autophagy (*In vivo*, Mice, i. p., 40 mg/kg, 28d, PBS buffer (negative control)) (Shang et al., 2024). However, this study lacked an active comparator group. Replication studies incorporating appropriate controls are warranted, and further investigation should explore additional mechanisms underlying the antitumor properties of PA in HCC. Currently, there are limited studies on the adverse reactions and toxicity of Physalin A. Before it can be used for treatment, comprehensive clinical trials, determination of effective doses, assessment of pharmacokinetic parameters, investigation of potential adverse reactions, and evaluation of interactions with other medications are required.

Salidroside is a major active substance in Rhodiola rosea L., which has been used as a hepatoprotective drug for decades (Khanna et al., 2017). In recent years, Salidroside has been found to possess a variety of pharmacological effects including anti-tumour, anti-inflammatory, anti-viral, anti-radiation, anti-oxidative stress and anti-fatigue. Studies have shown that Salidroside can inhibit HCC metastasis by suppressing the activation of the Notch1 signalling pathway (Lu et al., 2019), and also inhibit HCC viability and induce apoptosis by activating endoplasmic reticulum stress (Ding et al., 2020). Recent studies have found that Salidroside, an active metabolite derived from the rhizome of Rhodiola rosea (Zhang et al., 2021), diminishes the phosphorylation of PI3K and AKT proteins, enhances the expression of Bax, Caspase-3, and Caspase-9 proteins within the Wnt/β-catenin signalling pathway, reduces the expression levels of Bcl-2, LC3-1, and p62 proteins, and induces apoptosis in 97H cells by modulating mitochondrial function and autophagy (Jiang et al., 2023b). Recent research demonstrates that salidroside imposes a dual blockade at both M-phase and G1/S-phase transition in HCC cells, inducing irreversible cell cycle arrest. This mechanism likely underlies its critical role in significantly sensitizing HCC to 5-FU. Concordantly, the salidroside/5-FU combination demonstrated superior antitumor efficacy *in vivo* compared to 5-FU monotherapy (Sun et al., 2024). (*In vivo*, Mice, i. p., 50–100 mg/kg/day, 4weeks; Controls: 5-FU(positive control), phosphate-buffered saline (negative control)). However, the lack of toxicological studies on Salidroside limits its further research and application, and long-term toxicity studies and well-designed clinical trials are needed to provide a strong safety guarantee for its clinical application (Zhang et al., 2021).

Polygonum cuspidatum is a TCM that has been included in pharmacopoeia for a long time (Ke et al., 2023). One of its main metabolites, Polydatin, has been shown to possess various biological functions, such as protecting against ischaemia/reperfusion injury (Gao et al., 2016), congestive heart failure (Ling et al., 2016) and anticancer effects. Polydatin inhibited Wnt/β-catenin signalling activity in a concentration-dependent manner, upregulated the protein expression of downstream targets such as caspase-3, caspase-9, and Bax, and downregulated Bcl-2 protein expression, thereby inducing apoptosis in HepG2 and SMMC-7721 cells (*In vivo*, Mice, i. p., 25–100 mg/kg, 20 days, PBS buffer (negative control)) (Jiao et al., 2018). Animal models have demonstrated that Polydatin modulates HCC progression through multiple mechanisms; subsequent studies in humans are warranted to investigate its potential mechanisms of action for ameliorating HCC.

## 7 Crude extracts of single botanical drugs modulate Wnt/β-catenin signaling pathway to influence the progression of HCC

**FIGURE 4:**
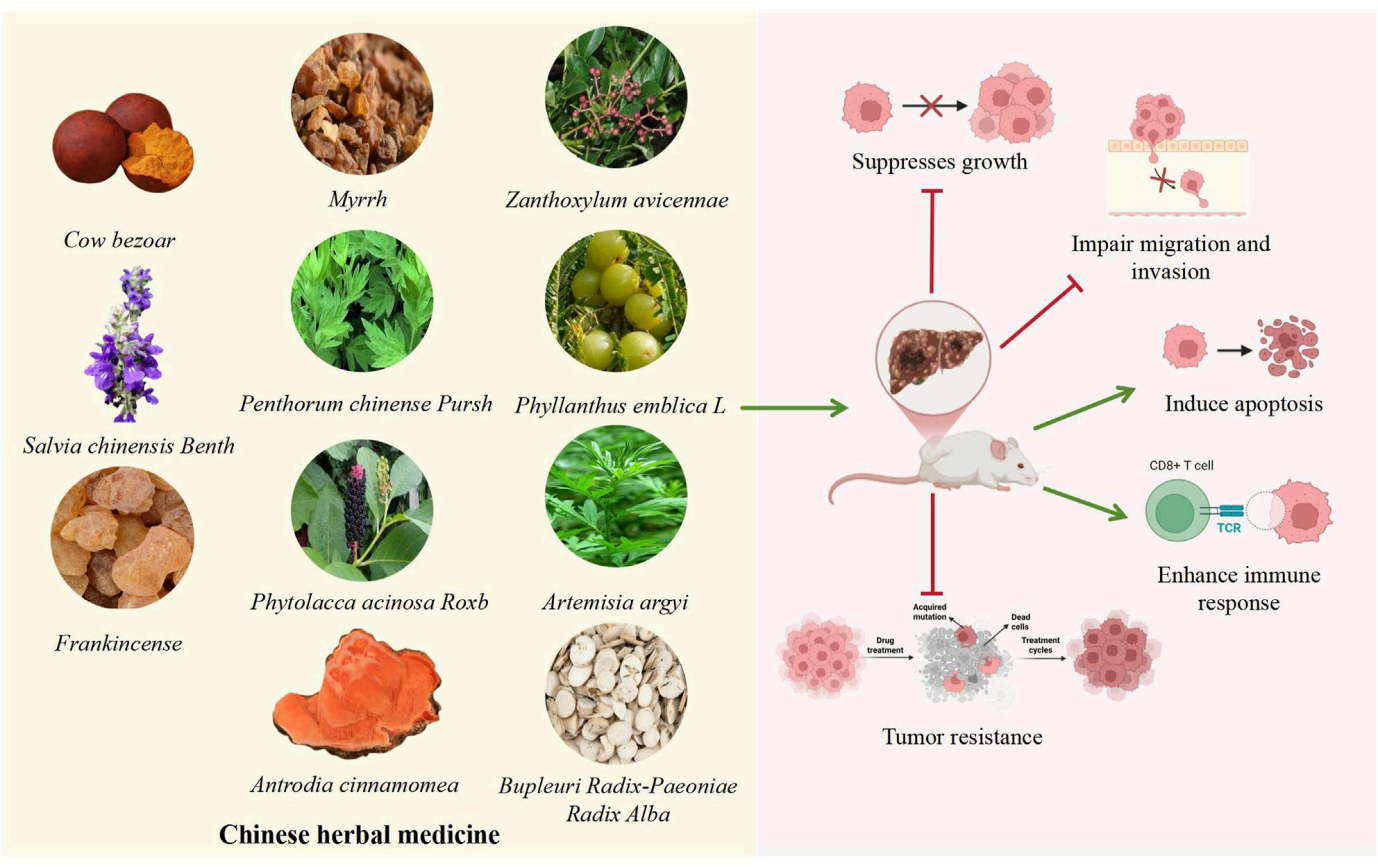
Potential Mechanisms of Crude extracts of single botanical drugs in HCC. The TCM shown in the left box can affect HCC. The specific mechanism of action shown in the diagram on the right: the green arrow represents the promoting effect, and the red arrow represents the inhibitory effect. These TCM may affect the progression of HCC by inhibiting proliferation, migration, and tumor resistance, promoting apoptosis and immune response.

Traditional Chinese medicine has been utilized for millennia and may serve as a therapeutic option for HCC (Li J. J. et al., 2021; Hu et al., 2013). Multiple studies have demonstrated that crude extracts of single botanical drugs from TCM can suppress tumer growth, impede cell migration and invasion, cause apoptosis, and treat HCC via the Wnt/β-catenin signalling pathway (Sun et al., 2018; Hu et al., 2013) (As shown in Table 4). This is illustrated in Figure 4.

**TABLE 4:** Crude extracts of single botanical drugs target the Wnt/β-catenin signalling pathway to treat HCC.

Metabolite name	Source	Extraction method	Cell	Laboratory animal	Injection method	Dose	Experimental period	Control method	Function	Mechanism of action	References
Cow bezoar	Cow bezoar	Ultrasonic extraction method	HepG2 cells	Mice	p.o	45.5–227.5mg/(kg/d)	15d	Saline (negative control)	Suppresses growth of HCC	Wnt/β-catenin, M2-TAM Polarization↓	Huang et al. (2024)
Phytolacca acinosa Roxb	Phytolacca acinosa Roxb	—	HepG2 and HCCLM3 cells	Mice	i.p	50–100 mg/kg	21 d	Saline (negative control)	Suppresses growth and induces apoptosis	Caspase-3↑, PI3K/Akt/MMP9, Bcl-2↓	Liu et al. (2024b)
Antrodia cinnamomea	Antrodia cinnamomea	Ultrasonic extraction method	HepG2, Huh-7 and SMMC-7721 cells	Mice	i.g	200–400 mg/kg/d	9 d	Saline (negative control)	Suppresses growth of HCC	PIK3CA/p-AKT↓, caspase 8 ↑	Zhang et al. (2022)
Salvia chinensis Benth	Salvia chinensis Benth	Decoction method	PLC/PRF/5 cells	Mice	p.o	100 mg/kg/d	5weeks	—	Suppresses growth and cell cycle progression and distant	Wnt/β-catenin, Wilms’ tumor 1 and β-catenin ↓	Wang et al. (2017)
Zanthoxylum avicennae	Zanthoxylum avicennae	Solvent extraction method	HA22T cells	Mice	p.o	20–40 mg/kg	2 weeks	—	Dose-dependently inhibits HCC cell metastasis signals	Activates PP2A, promotes β-catenin degradation, Wnt/β-catenin↓	Wu et al. (2017)
Phyllanthus emblica L	Phyllanthus emblica L	—	BEL-7404 and HepG2	Mice	i.p	0.15–0.6 g/kg	14d	Saline (negative control), 5-fluorouracil (positive control)	Reduces tumor weight and volume, repairs necrotic areas, and induces apoptosis in HCC cells	PI3K/AKT, Wnt/β-catenin↓	Tang et al. (2022a)
Frankincense and myrrh	Frankincense and myrrh	Decoction method	HCC-LM3 and Hepa1-6 cells	Mice	i.g	1.5and 3.0 g/kg/d	14d	Saline (negative control)	Inhibits the migration and invasion of HCC cells and inhibits EMT	E-cadherin↑PI3K/Akt , MAPK, N-cadherin, Disheveled 2, Wnt/β-catenin and EMT↓	Lu et al. (2023), Zheng et al. (2022)
Artemisia argyi Essential Oil	Artemisia argyi H.Lév. and Vaniot	Maceration method	HepG2 and SMMC-7721	Mice	i.p	57.5–230 mg/kg/d	21d	Sorafenib (positive control)	Suppresses growth, migration and invasionof HCC	DEPDC1, Wnt1, β-catenin, EMT和Wnt/β-catenin ↓	Li et al. (2021d)
Bupleuri Radix-Paeoniae Radix Alba	Bupleuri Radix-Paeoniae Radix Alba	Decoction method	MHcc-97H, HepG2 and Hep3B cells	Mice	i.g	1.0–4.0 g/kg	28d	Saline (negative control)	Suppresses growth and induces apoptosis	miR-1297, Akt, PI3K↓, PTEN, caspase-3/caspase-9 and Bax↑	Ding et al. (2024), Zhang et al. (2024b)

Cow bezoar (CB), a traditional Chinese drug and an metabolite of TCM Pien Tze Huang (Zhao, 2024), Research has found that the combination of CB and musk can induce apoptosis in liver cancer cells such as SMMC-7721 and HepG2 (Yu Z. J. et al., 2020). Previous studies have demonstrated the mechanisms of CB in liver cancer, including enhancing macrophage phagocytosis, inhibiting the secretion of pro-inflammatory cytokines, and alleviating liver inflammation and damage (Xiang et al., 2023). Additionally, Cow bezoar can inhibit the proliferation of HepG2 cells by modulating the Wnt/β-catenin signalling pathway, hence inhibiting the polarization of M2 tumour-associated macrophages (M2-TAM) (*In vivo*, Mice, p. o., 45.5–227.5 mg/kg, 7 days, Saline (negative control)). However, the 227.5 mg/kg dose approaches the maximum tolerated dose in mice. Definitive safety assessment of this dosage in animal studies and concomitant cytotoxicity studies are warranted.

Wnt1 is overexpressed in human HCC, activating Wnt/β-catenin signalling in a β-catenin-dependent way, hence boosting the proliferation and invasion of HCC cells; its elevated expression correlates with a reduced survival rate (Dropmann et al., 2024). Salvia chinensis Benth was extensively documented in the Compendium of Materia Medica (Ming Dynasty, 1590 AD). Salvia chinensis Benth is commonly used to treat bone pain and abscesses (Wang Q. et al., 2018). Previous studies have shown that Salvia chinensis Benth exhibits anticancer effects against breast cancer, stomach cancer, nasopharyngeal cancer, lung cancer, colon cancer, liver cancer, and pancreatic cancer (Wang K. N. et al., 2022; Zheng et al., 2023). Additionally, studies have found that Salvia chinensis Benth suppresses Wnt/β-catenin activity by modulating Wnt1 to reduce β-catenin transcription in HCC cells, impeding the *in vitro* proliferation of PLC cells and obstructing the cell cycle (*In vivo*, Mice, p. o., 100 mg/kg/d, 5 weeks). However, this study lacked both negative and active comparator groups. Replication studies incorporating rigorously designed controls are warranted to validate the robustness of the conclusions.

Recent studies indicate that almost 90% of mortality associated with HCC is due to metastasis. EMT is pivotal in the initial phases of metastasis, during which cells relinquish their adhesive properties and acquire motility to migrate to adjacent or distant organs (Giannelli et al., 2016). Consequently, Frankincense and myrrh can impede the invasion and metastasis of HCC-LM3 cells by enhancing the expression of the EMT marker E-cadherin, diminishing the protein and nuclear staining levels of vimentin, N-cadherin, Disheveled 2, and β-catenin, while also inhibiting the activation of the Wnt/β-catenin signalling pathway and EMT (Lu et al., 2023). The material basis and synergistic mechanisms of the frankincense-myrrh combination are still in their infancy, primarily through the separation and screening of chemical metabolites to identify simple bioactive metabolites (Cao B. et al., 2019). However, this approach is not suitable for the complex composition and multi-target synergistic effects of the frankincense-myrrh combination. Therefore, it is recommended that future studies combine new methods such as systems biology and metabolomics to investigate the chemical and pharmacological properties of frankincense and myrrh, thereby elucidating the material basis and mechanisms of action of frankincense, myrrh, and their combination. Furthermore, it has been established that PP2A targets the Axin-mediated degradation of the β-catenin complex (Wu et al., 2014). Zanthoxylum avicennae facilitates its interaction with β-catenin through the activation of PP2A, which expedites the degradation of β-catenin protein, markedly diminishing its levels in both the nucleus and cytoplasm and obstructing the propagation of metastasis signals in highly metastatic HA22T cells (*In vivo*, Mice,p.o., 20–40 mg/kg, 2 weeks) (Wu et al., 2017). However, this study lacked an active comparator group. Replication studies incorporating a clinically relevant positive control agent are warranted to validate the accuracy and reliability of the conclusions. And Zanthoxylum avicennae is easily confused with Zanthoxylum nitidum (Roxb.) DC., Zanthoxylum scandens, and Toddalia asiatica in the herbal medicine market (Wang X. et al., 2022). Therefore, it is necessary to study the quality markers of Zanthoxylum avicennae to enable rapid differentiation and strict quality control.

Artemisia argyi Lévl. et Vant. is a well-known TCM for treating dysmenorrhea, abdominal pain, and inflammation (Hu W. J. et al., 2024). Recent studies have shown that it has antioxidant, anticancer, anti-inflammatory, immunomodulatory, and antibacterial properties (Hu W. J. et al., 2024). A variety of phytochemicals have been identified in Artemisia, including essential oils, flavonoids, organic acids, and terpenes, which can inhibit the metastasis of various cancers (Li S. et al., 2018). *In vivo* studies demonstrated that sorafenib treatment suppressed tumor metastasis in mice but induced significant body weight loss, potentially attributable to systemic toxicity. In contrast, AAEO treatment significantly reduced both the number and size of pulmonary metastatic nodules compared to the sorafenib group, without inducing body weight loss throughout the treatment period. Further studies have shown that Artemisia argyi essential oil effectively inhibits the metastasis of HCC to lung, brain, and femoral tissue by inhibiting the Wnt/β-catenin signaling pathway and EMT to inhibit the expression of DEPDC1, and exhibits low toxicity and concentration dependence (*In vivo*, Mice, i. p., 57.5–230 mg/kg/d, 21day, Sorafenib (positive control)) (Li Y. et al., 2021). However, the 230 mg/kg dosage approaches the maximum tolerated dose in mice. Concomitant cytotoxicity studies and definitive safety assessments of this dose in animal models are warranted. The composition and content of AAEO vary significantly, influenced by factors such as origin, harvest time, aging methods, and extraction techniques. Therefore, it is essential to identify relatively consistent characteristics amidst these variations and establish scientific quality control standards for AAEO through comprehensive experimental studies.

## 8 Chinese herbal formulas modulate Wnt/β-catenin signaling pathway to influence the progression of HCC

According to TCM doctrine, Chinese herbal formulas comprise diverse metabolites. Each formula exhibits distinct effects and is defined by numerous pathways, diverse targets, and minimal adverse reactions. It can proficiently modulate the tumour immune microenvironment and enhance effectiveness (Zhang et al., 2020). It is clinically significant for mitigating side responses, enhancing patient survival rates and quality of life, and suppressing tumour growth (Yuan et al., 2020; Pu et al., 2022).

### 8.1 Suppresses growth of HCC

Baobao Dan has functions such as clearing heat, removing dampness, promoting blood circulation, detoxifying, and alleviating pain (Cheng et al., 2021) and has been approved by the China Food and Drug Administration (CFDA) as an alternative and adjunctive drug for the treatment of various cancers. It is composed of Bovis Calculus, Snake Gall, Notoginseng Radix et Rhizoma, Moschus chrysogaster, Saigae Tataricae Cornu, and Margarita. (Yu et al., 2019). Additionally, studies have found that Babao Dan inhibits the expression of Wnt downstream target genes Axin-2, cyclin D1, and c-myc, along with the HCC stem cell marker Epithelial cell adhesion molecule (EpCAM), in a dose-dependent manner. It obstructs the nuclear translocation of β-catenin, thereby inhibiting the proliferation of well-differentiated HepG2 and HEK293 cancer cells while also diminishing the proportion of EpCAM + CSCs associated with cancer stemness and tumour progression (*In vivo*, Mice, i. g., 0.12–0.24 g/kg/2d, 26 days, PBS (negative control)) (Xie X. et al., 2021). However, this study lacked an active comparator group. Replication studies incorporating a clinically relevant positive control agent are warranted to validate the accuracy and reliability of the conclusions. Research has identified N-Gin R1, Gin Re, Gin Rg1, Gin Rb1, GCA, Gin Rd, CA, TCA, CDCA, and DCA as potential chemical markers for BBD quality control. These markers can assist in the quantitative analysis and quality control of BBD, laying the foundation for its clinical application and further research (Cheng et al., 2021).

Jiedu Xiaozheng Yin is a Chinese herbal formula composed of Scleromitrion diffusum (Willd.) R.J.Wang, Sophora flavescens Aiton, Cremastrae Pseudobulbus Pleiones Pseudobulbus and is used as a heat-clearing and detoxicating adjuvant therapy for HCC. (Liu et al., 2017). Research has shown that Jiedu Xiaozheng Yin can target cancer stem cells in colorectal cancer by regulating the Wnt signaling pathway (Feng et al., 2025). Additionally, studies have found that Jiedu Xiaozheng Yin induces apoptosis through a mitochondria-mediated pathway, thereby inhibiting the proliferation of liver cancer cells (Cao et al., 2015), and can also inhibit the proliferation of HCC cells by inducing G0/G1 phase arrest in both *in vivo* and *in vitro* experiments. Randomised controlled trials have shown that Jiedu Xiaozheng Yin has a certain effect on patients with stage III primary liver cancer during the perioperative period, improving immune function, reducing recurrence rates, and increasing cumulative survival rates (Chen et al., 2014; Chen et al., 2005). (Clinical trial, 72 patients,p.o.,2 years) Moreover, Jiedu Xiaozheng Yin not only facilitates the translocation of β-catenin from the cytoplasm and nucleus to the cell membrane but also suppresses the production of PCNA, c-myc, cyclin D1, and Bmi1, thus reducing the proliferation of Huh7 cells (*In vivo*, Mice, p. o. 0.13 g/kg/day, 3 weeks) (Liu et al., 2017; Chen et al., 2014). However, this study lacked an active comparator group and evaluated Jiedu Xiaozheng Yin at only 0.13 g/kg without dose-ranging exploration. Replication studies incorporating a clinically relevant positive control agent and multi-dose investigations of Jiedu Xiaozheng Yin are warranted to validate the accuracy and reliability of the conclusions and establish its minimum effective dose (MED) against HCC.

Gehua Jiecheng Decoction (GHJCD) originates from the theory of spleen and stomach function proposed by Li Dongyuan, one of the four great medical experts of the Jin-Yuan period. It is composed of Prunus armeniaca L., Aquilaria malaccensis Lam., Citrus × limon (L.) Osbeck, Withania somnifera (L.) Dunal, Polyporus umbellatus, Poria cocos, Fried Shenqu, Alisma plantago-aquatica L., Zingiber officinale Roscoe, Atractylodes macrocephala Koidz., Nutmeg kernel, Pueraria montana var. thomsonii (Benth.) M.R.Almeida, and Wurfbainia villosa (Lour.) Škorničk. and A.D.Poulsen, a total of 13 TCMs.In China, GHJCD is commonly used to treat and liver cirrhosis and liver damage caused by alcohol consumption (Yang, 2007). Research has found that GHJCD can also inhibit HCC cells in subcutaneous transplantation and *in situ* liver transplantation in mice. In particular GHJCD suppresses the production of GST-Pi and PCNA in hepatic tissue by inhibiting the Wnt/β-catenin signalling pathway, consequently impeding the proliferation of tumour stem cells and correcting HCC precursor lesions (*In vivo*, Mice, p. o., 10 mL/kg, 23 weeks, Sterile water (negative control)) (Cheng et al., 2020). However, this study lacked an active comparator group. Replication studies incorporating a clinically relevant positive control agent are warranted to validate current findings, and clinical RCTs remain essential to establish the therapeutic efficacy of GHJCD.

Yiqi Liangxue Jiedu Prescription (YLJP), which has been authorised by the national patent (No. ZL202110889980.5) for the prevention and treatment of HCC, can inhibit the classical Wnt pathway by decreasing the expression of Wnt1 and β-catenin, and inhibit the value-adding and cancerous transformation of HOC (Hepatocellular Originated Pre-cancer Cells), thus suppressing the occurrence of HCC. YLJP is composed of Adenophora stricta Miq, Ophiopogon japonicus (Thunb.) Ker Gawl., Codonopsis pilosula (Franch.) Nannf., Astragalus mongholicus Bunge, Scleromitrion diffusum (Willd.) R.J.Wang, Paris polyphylla Sm., Atractylodes macrocephala Koidz., Wolfiporia extensa Ginns, Angelica sinensis (Oliv.) Diels, and Rehmannia glutinosa (Gaertn.) Libosch. ex DC. In addition, the clinical study found that the 1-year HCC incidence rate of patients treated with YLJP was significantly lower than that of the Western medicine group treated with ETV and TAF antiviral therapy. (Clinical trial, 17 patients, p. o., 3 months, ETV and TAF (positive control)) (Liang et al., 2024). However, this study was limited by a small sample size (n = 17) and the absence of a placebo control. Validation in adequately powered RCTs is required to establish the scientific validity and reliability of these findings (Other examples are shown in Table 5).

**TABLE 5:** Chinese herbal formulas targeting the Wnt/β-catenin signalling pathway suppress the growth of HCC.

Name composition	Composition	Extraction method	Cell	Laboratory animal	Injection method	Dose	Experimental period	Control method	Function	Extraction method	Theory	References
Liuweiwuling Tablet	Schisandra chinensis (Turcz.) Baill., Ligustrum lucidum W.T.Aiton, Forsythia suspensa (Thunb.) Vahl, Curcuma zedoaria (Christm.) Rosc., Sonchus wightianus DC. And Ganoderma lucidum (Curtis) P. Karst	—	Huh7, HepG2, LX-2 and LO2 cells	rat	p.o	0.81–1.62 g/kg	18 weeks	—	Suppresses growth, including the initiation of apoptosis and a decrease in the expression of stemness genes	PI3K mRNA, PI3K, AKT, IKKα/β, IκBα, p65, Smad3 and Smad2↓	Nourishes yin and blood, clears heat and detoxifies	Chen et al. (2024)
Jiedu Xiaozheng Yin	Scleromitrion diffusum (Willd.) R.J.Wang, Sophora flavescens Aiton, Prunella vulgaris L. and Cremastra appendiculata (D.Don) Makino	Solvent extraction method	PLC/PRF/5 and Huh7 cells	Mice	i.g	0.13 g/kg/d	3 weeks	Saline (negative control)	Suppresses tumer growth and induces G0/G1 phase block to inhibit HCC cell proliferation	PCNA, β-catenin downstream genes such as Bmi1, Wnt/β-catenin ↓, p16INK4A ↑	Clears away heat and toxins, and resolves stagnation	Chen et al. (2014)
Jiedu Recipe	Actinidia valvata Dunn, Salviae Chinensia Herba, Cremastra appendiculata (D.Don) Makino and Galli Gigeriae Endothelium Corneum	Solvent extraction method	SMMC-7721 and Huh7 cells	Mice	i.g	0.2 mL	4 weeks	Distilled water (negative control)	Suppresses growth, migration and invasion	E-cadherin↑, HIF-1α mRNA, Wnt/β-catenin downstream target genes, β-catenin/p-GSK-3β↓	Clears away heat and toxins, resolves phlegm and stops malaria, relieves pain	Guo et al. (2023)
Babao Dan	Bovis Calculus, Abelmoschus moschatus Medik., Panax notoginseng (Burkill) F.H.Chen, Snake Gall, Saigae Tataricae Cornu and Citrus japonica Thunb	Solvent extraction method	HepG2 and HEK293 cells	Mice	i.g	0.12–0.24 g/kg/2d	26 days	PBS (negative control)	Suppresses growth of HCC	Wnt3a, Wnt/β-catenin, GSK-3β, Wnt downstream target genes, EpCAM↓	clearing away heat and toxins, resolving phlegm and promoting circulation of Qi, harmonizing the stomach and stopping vomiting, and relieving jaundice and pain	Xie et al. (2021b)
Huatan Sanjie Granules	Cremastra appendiculata (D.Don) Makino, Arisaema erubescens (Wall.) Schott, Curcuma longa L., Curcuma aromatica Salisb., Bolbostemma paniculatum (Maxim.) Franquet and Fritillaria thunbergii Miq	—	HepG2, Hep3B and Huh-7 cells	Human	p.o	—	2 months	Western medicine treatment (positive control)	Suppresses growth, induce apoptosis and cell cycle arrest	Bcl-2 ↑,Bax/Caspase-9, PI3K-Akt/MAPK ↓	Detoxify, resolve phlegm and reduce swelling	Yuan et al. (2023)
Zuojin Pills	Coptis chinensis Franch. and Tetradium ruticarpum (A.Juss.) T.G.Hartley	Decoction method	HCC HepG2, MHCC97L, PLC/PRF/5 and HLE cells	Mice	i.g	400 mg/kg/2 d	4 weeks	PBS (negative control)	Suppresses tumer growth	pMAPK1, PIK3CA 和 EGFR↓CCND1↓NFKBIA↑	Soothes the liver and clears away heat, harmonizes the stomach and reduces counterflow	Guo et al. (2019)
Gehua Jiecheng Decoction	Prunus armeniaca L., Aquilaria malaccensis Lam., Citrus × limon (L.) Osbeck, Withania somnifera (L.) Dunal, Polyporus umbellatus, Poria cocos, Fried Shenqu, Alisma plantago-aquatica L., Zingiber officinale Roscoe, Atractylodes macrocephala Koidz., Nutmeg kernel, and Pueraria montana var. thomsonii (Benth.) M.R.Almeida, Wurfbainia villosa (Lour.) Škorničk. and A.D.Poulsen	Decoction method	—	Mice	p.o	15 μg/mL	23 weeks	Sterile water (negative control)	Suppresses growth, and inhibits the angiogenic capacity of hepatocellular carcinoma	APC↑, WNT1/β-catenin, Frz-7, IL-6, IL-10, TNF-α↓	Eliminates dampness and warms the middle Jiao	Cheng et al. (2020)
Qizhu anticancer prescription	Galli Gigerii Endothelium Corneum, Astragalus mongholicus Bunge, Curcuma longa L., Atractylodes macrocephala Koidz., Coix lacryma-jobi var. ma-yuen (Rom.Caill.) Stapf, Paeonia lactiflora Pall., Bupleurum chinense DC., Scleromitrion diffusum (Willd.) R.J.Wang. And Glycyrrhiza glabra L	Decoction method	Huh7, MHCC-97H, AML12 and Miha cell lines	Mice	p.o	3.51–7.02 g/kg	14 weeks	Saline (negative control), PD1 (positive control)	Suppresses growth of HCC	p21, WNT2, CXCL14 ↑, Ki67 ↓	Detoxifies and strengthens the spleen and liver, and fights cancer	Hu et al. (2024c)
Hu gan Pills	Bupleurum chinense DC., Artemisia scoparia Waldst. and Kit., Isatis tinctoria L., Schisandra chinensis (Turcz.) Baill., Suis Fellis Pulvis, and Testa Vignae Radiate	Solvent extraction method	H22 and HepG2 cells	Mice	i.g	100–300 μg/20 g	7 days	Water (negative control)	Suppresses tumor growth	caspae 3, LC3II and beclin1 ↑, cyclinE1, CDK2 and CDK4↓	Soothes the liver and regulates qi, protects the liver and lowers enzymes	Gao et al. (2019)

### 8.2 Impair HCC cellular migration and invasion

Research indicates that Chinese herbal formulas can enhance the pre-metastatic microenvironment of HCC and inhibit the metastatic potential of HCC cells by modulating the Wnt/β-catenin signalling system (As shown in Table 6).

**TABLE 6:** Chinese herbal formulas targeting the Wnt/β-catenin signalling pathway impair the invasion and migration of HCC cells.

Name composition	Composition	Extraction method	Cell	Laboratory animal	Injection method	Dose	Experimental period	Control method	Function	Extraction method	Theory	References
Compound Phyllanthus urinaria L	Phyllanthus Urinaria L., Astragalus mongholicus Bunge, Curcuma longa L., Scutellaria barbata D.Don and Cremastra appendiculata (D.Don) Makino	—	HepG2, SMMC-7721 and Huh-7 cells	Mice	p.o	300–625 mg/kg	34d	Saline (negative control)	Inhibits the metastasis of HBV-related HCC	Ubiquitination and proteasome degradation of β-catenin↑	Clears away heat and toxins, dispels dampness and benefits the gallbladder	Huang et al. (2021c)
Compound Kushen Injection	Sophora flavescens Aiton and Heterosmilacis. Commelina tuberosa L	—	SMMC-7721 cells	Mice	p.o	1.5–3 mL/kg	3 weeks	5-FU (positive control)	Inhibits cell migration and metastasis and EMT	β-catenin/c-Myc↓	Clears away heat and dampness, cools the blood to detoxify, disperses lumps and relieve pain	Wang et al. (2021c)
			—	—	—	—	—	—	Inhibit cell migration	p53,PI3K-Akt↓		He et al. (2020)
Ba Bao Dan	Bovis Calculus, Abelmoschus moschatus Medik., Panax notoginseng (Burkill) F.H.Chen, Snake Gall, Saigae Tataricae Cornu and Citrus japonica Thunb	Ultrasonic extraction method	HepG2 and Hep1-6 cells	Mice	i.g	0.125–0.5 g/kg	2 weeks	Saline (negative control)	Inhibits the development and metastasis of HCC cells	PI3K/AKT/mTOR↓	Clears away heat and toxins, relieves pain	Liu et al. (2024c)
Jiedu Granule formula	Actinidia valvata Dunn, Salvia chinensis Benth, Pseudobulbus Cremastrae seu Pleiones and the gizzard membrane of Gallus *domesticus* Brisson	—	MHCC-97H cells	Mice	i.g	12.74–63.7 g/kg/d	2 weeks	Saline (negative control)	Inhibits EMT and hepatoma cell migration	CSF1/PI3K/Akt↓	Clears away heat and toxic materials, promotes blood circulation and relieves pain	Liu et al. (2024d)
Biejiajian Pills	Colla Carapacis Trionycis, Asini Corii Colla, Vespae Nidus, Armadillidium veluchieuse, Eupolyphaga, Catharsius molossus L., Bupleurum chinense DC., Scutellaria baicalensis Georgi, Pinellia ternata (Thunb.) Breit., Codonopsis pilosula (Franch.) Nannf., Zingiberis Rhizoma, Magnoliae Officinalis Cortex, Cinnamomum cassia (L.) C. Presl, Paeoniae Radix Alba, Belamcanda chinensis (L.) Redouté, Persicae Semen, Paeonia suffruticosa Andr, Rheum palmatum L, Campsis grandiflora (Thunb.) Schum, Lepidium apetalum Willd, Pyrrosia lingua (Thunb.) Farwell and Dianthus superbus L	—	HepG2 cells	Mice	i.g	10 mL/kg,2times/d	3d	Saline (negative control)	Suppresses tumor growth, adhesion and metastasis	DKK-1, Wnt/β-catenin↓	Promotes blood circulation and removes blood stasis, detoxifies and resolves lumps	Wen et al. (2014)
Fuzheng Jiedu Xiaoji formulation	Codonopsis pilosula (Franch.) Nannf.,Astragalus mongholicus Bunge, Atractylodes macrocephala Koidz.,Poria cocos, Adenophora stricta Miq., Ophiopogon japonicus (Thunb.) Ker Gawl., Angelica sinensis (Oliv.) Diels,Rehmannia glutinosa (Gaertn.) Libosch. ex DC.,Paris polyphylla Sm., Curcuma phaeocaulis Valeton and Pinellia ternata (Thunb.) Makino	—	MHCC97H and BEL7402 cells	Mice	i.g	64.6 mg/12 h	2 weeks	Saline (negative control)	Suppresses growth and migration of HCC	p-AKT (Thr308/Ser473), CyclinD1↓p27 and p21↑	Strengthens the immune system, detoxifies, relieves pain	Yang et al. (2021a)

Biejiajian Pills (BJJP), from ‘JinGuiYaoLue’, has the effects of benefiting qi and nourishing blood, activating blood circulation and removing blood stasis, and detoxifying and dispersing toxins and nodules. BJJP is primarily composed of Trionycis Carapax, Donkey-hide Gelatin, *Armadillidium vulgare*, Nidus Vespae, Eupolyphaga seu Steleophaga, Catharsius molossus, Scutellaria baicalensis Georgi, and Ginseng Radix et Rhizoma and it is now widely used in the treatment of hepatocellular carcinoma, cirrhosis and liver fibrosis (An et al., 2018). *In vivo* experiments revealed that BJJP dose-dependently inhibited hepatic fibrosis and reduced HCCcells and inflammatory cell infiltration in rats by enhancing antioxidant capacity and down-regulating inflammation-related pathways. Specifically, BJJP reduced the expression of NLRP3, apoptosis-associated speck-like protein (ASC), caspase-1, pro-IL-1β, pro-IL-18, IL-1β, and IL-18 in the livers of Den-treated rats (Feng et al., 2020). (*In vivo*,Rat,i.g., 10 mL/kg,2times/d, Saline (negative control)) Given the multi-component nature of BJJP and its potential polypharmacology, rigorous mechanistic studies pose significant challenges. This investigation employed integrated dose-response relationship analysis to identify dose-dependent therapeutic effects and robustly associate them with pathway-level mechanisms. Subsequent molecular docking experiments are warranted to further elucidate the underlying mechanisms of action. *In vitro* experiments revealed that BJJP-treated serum significantly reduced GSK-3β and β-catenin/TCF4 complex activity, thus reducing the expression of cytoplasmic and nuclear β-catenin proteins, DKK-1, CD44v6, VEGF, cell cycle protein D1 and MMP-2 proteins, and thus may effectively inhibit the invasiveness and migration of HCCcells (Feng et al., 2020; Sun et al., 2014).

Clinical studies have found that Compound Phyllanthus urinaria L. (CPUL) inhibits the development of HCC by improving the immune system, reversing liver fibrosis, blocking the cell cycle, and inhibiting angiogenesis. The metabolites of CPUL included Phyllanthus urinaria L., Scutellaria barbata D.Don, Curcumae Longae Rhizoma, Astragalus mongholicus Bunge and Edible tulip. After a 24-month treatment period, the number of antibody positives was lower in the treatment group (1.08 ± 1.01) than in the control group (2.11 ± 1.12) (Tong et al., 2014). (Clinical trial, 102 patients, p. o., 3 years, Untreated patients (blank control))Meanwhile, *in vivo* experiments revealed that CP facilitates the autophagic degradation of Cav-1 and enhances AKT/GSK-3β-mediated β-catenin proteasomal degradation via ubiquitination, consequently diminishing the pro-metastatic influence of Cav-1 and inhibiting the metastasis of HBV-related HepG2 cells (*In vivo*, Mice, p. o., 300–625 mg/kg, 34 days, Saline (negative control)) (Huang D. et al., 2021). However, the concentration of the CPUL used in the current study is high (Geethangili and Ding, 2018), and there is a subsequent need to assess its absorption, distribution, metabolism, and excretion levels, and to conduct quality control and toxicological studies in order to develop it as a nutritional supplement or therapeutic agent.

Compound Kushen Injection (CKI), which consists of Sophora flavescens Aiton and Heterosmilacis japonica Kunth, is approved by the Chinese State Food and Drug Administration (CFDA) for the treatment of various types of solid tumours. Clinical studies have shown that CKIs have significant efficacy in HCC when used alone or in combination with radiotherapy and chemotherapy approaches to improve 1- and 2-year survival (Ma et al., 2016). There is also a systematic review showing that CKI in combination with TACE improves survival, ORR and quality of life and reduces adverse events in patients with HCC. However, the results should be interpreted with caution due to the low methodological quality of the included SRs.The clinical efficacy of CKI must be demonstrated in a large number of randomised controlled trials (Lu T. et al., 2024). *In vivo* experiments showed that CKI alleviated tumour-associated macrophage-mediated immunosuppression via TNFR1 and enhanced the susceptibility of HCCto sorafenib sensitisation (Yang Y. et al., 2020). Following administration of 1.5 and 3 mL/kg CKI, gross observation revealed reduced or absent nodularity on the hepatic surface in rats. The 3 mL/kg CKI regimen demonstrated comparable efficacy to the 75 mg/kg 5-FU positive control group. Concurrent studies find that CKI suppresses c-Myc expression by modulating the Wnt/β-catenin signalling pathway, affecting metabolic reprogramming and EMT in HCC, therefore hindering the invasion and metastasis of SMMC-7721 cells (*In vivo*, Mice, p. o., 1.5–3 mL/kg, 3 weeks, 5-FU (positive control)) (Wang K. X. et al., 2021). However, this study evaluated CKI exclusively at 3 mL/kg without dose-ranging investigation. Subsequent studies should delineate the concentration-dependent effects of CKI on HCC to establish its effective dose range.

The principal activators of the NF-κB pathway, IKKα and IKKβ, can engage with β-catenin, resulting in its phosphorylation and the subsequent positive regulation of the Wnt/β-catenin pathway (Oliva-Vilarnau et al., 2024). Sini-San was first recorded in Zhang Zhongjing’s ‘ShanghanLun’ in the Han Dynasty, and has the efficacy of relieving the liver and depression, regulating the spleen and stomach, and is commonly used in the treatment of depression and other liver qi stagnation evidence. It is composed of Bupleurum chinense DC., Paeonia lactiflora Pall., Citrus × aurantium L., and Glycyrrhiza uralensis Fisch (Zhu et al., 2023), and has a good therapeutic effect on non-alcoholic fatty liver disease, hepatitis liver injury, and hepatic fibrosis (Jiang M. et al., 2023). Research indicates that Sini-San obstructs NF-κB nuclear translocation, diminishes NF-κB pathway signalling and AP-1 activity expression, and suppresses Wnt/β-catenin signalling, consequently exhibiting anti-invasion and anti-migration actions on HepG2 cells (Lin et al., 2015). However, as an *in vitro* study, these findings provide only preliminary evidence of Sini-San’s ameliorative effects on HCC cells. Validation in biologically complex animal models or clinical studies remains essential to establish its therapeutic potential.

### 8.3 Induce apoptosis of HCC cells and prevent the occurrence of HCC

Diwu Yanggan capsule (DWYGC) is a patented herbal formula (Patent No. 201210580999.2), which has been approved for use as a drug by the Hubei Provincial Food and Drug Administration. The mixture includes five Chinese medicinal herbal extracts, whose proportions (w/w) are as follows: Rehmannia glutinosa (Gaertn.) DC., Artemisia scoparia Waldst. and Kitam., Curcuma longa L., Schisandra chinensis (Turcz.) Baill. and Glycyrrhiza uralensis Fisch. DC. (Zhao B. B. et al., 2015). Previous studies have demonstrated that DWYGC plays an important role in enhancing immune-modulating responses, improving liver injury, promote liver regeneration, exert virucidal activity, and suppress HCC growth (Ye et al., 2018). DWYGC was discovered to promote apoptosis in HCC cells by reducing the expression of Wnt-1, Wnt3, β-catenin, FZD2, and GSK3β, leading to a reduction in the expression products of downstream target genes c-myc, cyclin D1, and EpCAM protein (*In vivo*, Rat, p. o., 360 mg/kg, 22 days, Distilled water (negative control)). Furthermore, 2-AAF/PH expedited the activation of the Wnt/β-catenin signalling pathway during the initial phase of hepatic regeneration in animals but subsequently inactivated the system, diminishing the risk of HCC (Zhao B. B. et al., 2019; Zhao B. B. et al., 2015). Although some studies have found that the DWYGC has a therapeutic effect on HCC (Shi et al., 2024), its safety and potential side effects need to be further clarified when compared to other treatments for HCC.

Kangxianruangan granule (KXRGG) has the efficacy of helping correcting and resolving stasis, softening and dispersing hard lumps. Since the 1990s, it has been clinically used to treat patients with liver fibrosis as well as cirrhosis with good efficacy. It is a classical formula containing Artemisia capillaris Thunb., Salvia miltiorrhiza Bunge, Turtle shell, Panax notoginseng (Burkill) F.H.Chen, Prunus persica (L.) Batsch, Angelica sinensis (Oliv.) Diels, Curcumae Rhizoma, Parched pangolin scales, Ground beeltle, Atractylodes macrocephala Koidz., Coix seed and Astragalus mongholicus Bunge (Liu et al., 2018). Recent studies found that KXRGG markedly diminishes the expression of Wnt1, β-catenin, Cyclin D1, c-Myc, MMP-7, Axin-2, and EpCAM proteins, inhibits the conversion of HOCs into HCC cells, and offers novel insights into the anti-fibrosis-HCC mechanism of drugs via the Wnt/β-catenin signalling pathway (Tang W. et al., 2022). This provides a basic research basis for the prevention and treatment of HCC. However, due to the complexity of the drug-containing serum metabolites of KXRGG, it was not possible to determine the effects and mechanisms of the individual metabolites, which will be the focus of further research in the future.

Yangzheng Xiaojifang (YZXJF) is a Chinese herbal formula with anticancer effects, and studies have found it to be effective in the treatment of many types of cancers, including lung cancer, breast cancer, stomach cancer, and HCC. It is composed of 16 traditional Chinese medicines named Astragalus mongholicus Bunge, Fructus ligustri lucidi, Ginseng Radix et Rhizoma, Curcumae Rhizoma, Ganoderma, Gynostemma pentaphyllum (Thunb.) Makino, Atractylodes macrocephala Koidz., Scutellaria barbata D. Don, Scleromitrion diffusum (Willd.) R.J.Wang, Poria cocos, Eupolyphaga seu steleophaga, Galli gigeriae endothelium corneum, Mock strawberry herb, Bittersweet herb, Artemisiae Scopariae Herba, and Cynanchi paniculati Radix et Rhizoma (Zhang Y. et al., 2019). Clinical trials have found that YZXJF combined with conventional radiotherapy and chemotherapy can suppress the growth of HCC, enhance the immune function of patients with intermediate and advanced HCC, and improve the therapeutic efficacy and the quality of patient survival (Liqiong, 2019; Ling, 2021). (Clinical trial, 80 HCC patients, p. o., 4 capsules/time, 3 times/day, 1 month, Untreated patients (blank control)) *In vivo* and *in vitro* experiments have found that YZXJF treatment exhibits scattered cellular distribution and reduced tumour cell density, mitosis, and heterogeneity, and can inhibit the proliferation of HCC cells in a dose- and time-dependent manner, and inhibit colony formation, reduce invasive activity, decrease migration, and induce apoptosis. Meanwhile, network pharmacological studies have identified 141 potential drug targets and 170 pathways, including key targets such as TNF, TP53 and CASP3. In addition, its genetic targets are focused on HCC-related pathways, such as PI3K/Akt, AMPK, apoptosis and cancer pathways (*In vivo*, mice, p.o., 0.072–1.404 kg/d, Saline (negative control); OXA (positive control)) (Zhou et al., 2024).

Moreover, specific Chinese herbal formulas, for example,: Huatan Sanjie Granules (HSG), is composed of six CHMs, including Cremastrae Pseudobulbus Pleiones Pseudobulbus, Arisaema Cum Bile, Curcuma longa L., Curcumae Radix, Bolbostemmatis Rhizoma, and Fritillariae Thunbergii Bulbus. These six CHMs have strong capabilities of phlegm resolution, detoxification, and resolving masses. A cohort study showed that the median survival time of primary liver cancer patients taking HSG was 269 days, which was 23 days longer than that of the control group. In particular, the median survival time of Barcelona Clinic Liver Cancer stage C patients in the HSG group was 411 days, 137 days longer than in the control group (Yuan et al., 2023). (Clinical trial, 97 HCC patients, p. o., 2 months, Untreated patients (blank control)) However, this study did not further explore the key active therapeutic ingredients and their direct targets in HSG through the “Jun Chen Zuo” theory of TCM. Therefore, in future research, UPLC-MS/MS technology can be used to explore the specific active ingredients involved in HSG and validate the relevant protein targets through target binding validation techniques such as SPR, ITC, etc. (Other examples are shown in Table 7).

**TABLE 7:** Chinese herbal formulas targeting the Wnt/β-catenin signalling pathway induces apoptosis in HCC cells.

Name composition	Composition	Extraction method	Cell	Laboratory animal	Injection method	Dose	Experimental period	Control method	Function	Extraction method	Theory	References
Biejiajian Pills	Colla Carapacis Trionycis, Asini Corii Colla, Vespae Nidus, Armadillidium veluchieuse, Eupolyphaga, Catharsius molossus L., Bupleurum chinense DC., Scutellaria baicalensis Georgi, Pinellia ternata (Thunb.) Breit., Codonopsis pilosula (Franch.) Nannf., Zingiberis Rhizoma, Magnoliae Officinalis Cortex, Cinnamomum cassia (L.) C. Presl, Paeoniae Radix Alba, Belamcanda chinensis (L.) Redouté, Persicae Semen, Paeonia suffruticosa Andr, Rheum palmatum L, Campsis grandiflora (Thunb.) Schum, Lepidium apetalum Willd, Pyrrosia lingua (Thunb.) Farwell and Dianthus superbus L	—	Huh7 and BMSCs cells	—	—	—	—	—	Inhibits the vitality of CSCs and enhances apoptosis	miR-140↑, Wnt/β-catenin, Wnt3a, CD 24, CD133 and EpCAM↓	Promotes blood circulation and resolves blood stasis, softens lumps and disperses knots	Jingjing et al. (2021)
			Huh7cells	Mice	i.g	1.1 g/kg	8 weeks	BMSCs (positive control)	Accelerate apoptosis of HCC cells while suppresses tumer growth	miR-140↑, Wnt/β-cateni, Wnt3a, CD 24, CD133 and EpCAM↓		Jingjing et al. (2022)
			HepG2 cells	Mice	i.g	0.55–2.2 g/kg	30d	Normal saline (negative control)	Suppresses growth of HCC and promotes apoptosis of HCC cells	PCNA, β-catenin, Tbx3 and VEGF↓	Activates blood circulation and removes blood stasis, detoxifies and resolves lumps	Wen et al. (2016)
Aidi injection	cantharis (Mylabris phalerata Pallas), Astmgali Radix (Astragalus membranaceus (Fisch.) Bge), ginseng (Panax ginseng C. A. Meyer) and acanthopanax root (Acanthopanax senticosus (Rupr. et Maxim.) Harms)	Ultrasonic extraction method	HepG2 and PLC/PRF/5 cells	Mice	i.p	1.5–3.0 g/kg/d	12 d	Distilled water (negative control)	Induces apoptosis in HCCcells	p-PI3k, Bcl-xL, p-p38, p-JNK and Bim ↑, MMP↓	Clears away heat and toxins, resolves blood stasis and disperses lumps	Lan et al. (2021)
Diwu Yanggan Modulates	Rehmannia glutinosa (Gaertn.) Libosch. ex DC., Curcuma longa L., Schisandra chinensis (Turcz.) Baill., Artemisia scoparia Waldst. and Kit. and Glycyrrhiza glabra L	—	—	Rat	p.o	10mL/kg	22d	Distilled water (negative control)	Suppresses growth of HCC	Wnt/β-catenin↓ Thy1.1, GSK3β/FZD2, downstream target gene expression products↑	Anti-inflammatory and liver-protecting, detoxifying and unblocking collaterals, clearing heat and dampness, tonifying the kidney and nourishing the liver	Zhao et al. (2019a)
YangzhengXiaoji capsule	Ligustrum lucidum W.T.Aiton, Panax ginseng C.A.Mey., Curcuma aromatica Salisb., Atractylodes macrocephala Koidz., Vincetoxicum mukdenense Kitag., Tubiechong and Jineijin	Decoction method	MHCC97H, HCCLM3, HepG2 and Huh7 cells	Mice	p.o	0.351–1.404 kg/d	28 d	Saline (negative control); OXA (positive control)	Suppresses growth, invasion and migration of HCC; induces apoptosis *in vitro*	p53/Caspase-3, Bax↑, Bcl-2, p-PI3K/PI3K and p-Akt/Akt↓	Strengthens the spleen and kidney, resolves blood stasis and detoxifies	Zhou et al. (2024)
Bushen-Jianpi Decoction	Rehmannia glutinosa (Gaertn.) DC,Cornus officinalis Siebold and Zucc, Dioscorea oppositifolia L, Panax ginseng C.A. Mey, Atractylodes macrocephala Koidz, Poria cocos (Schw.) Wolf, Alisma plantago-aquatica subsp. orientale (Sam.) Sam,Paeonia suffruticosa Andrews, and Glycyrrhiza uralensis Fisch. ex DC.	Decoction method	HepG2 cells	Mice	i.g	5.7 g/kg	15 d	Distilled water (negative control), 5-FU (positive control)	Induces apoptosis	PI3K, Bcl-xL/BAD, p-Akt and p-mTOR ↓; p53, CASP3 ↑	Strengthens the spleen and kidney, nourishes the qi and nourishes the yin	Wu et al. (2019)
Sinikangai fang	Scleromitrion diffusum (Willd.) R.J.Wang, Eupolyphaga sinensis Walker, Scutellaria barbata D. Don, Solanum nigrum L., Akebia quinata (Thunb. ex Houtt.) Decne., Bupleurum chinense DC., Paeonia lactiflora Pall., Citrus aurantium L., Glycyrrhiza uralensis Fisch., Codonopsis pilosula (Franch.) Nannf., Atractylodes macrocephala Koidz., Coix lacryma-jobi Lvar. Mayuen (Roman.) Stapf, Poria cocos (Schw.) Wolf, Prunus persica (L.) Batsch, Cremastra appendiculata (D. Don) Makino.	Decoction method	HCCLM3 and MHCC97H cells	Mice	p.o	15.3–61.2 g/kg	15d	Saline (negative control); Sorafenib (positive control)	Suppresses growth, migration, invasion and induces apoptosis in tumor	cleaved-caspase-9, cleaved-caspase-3 and Bax↑, Bcl-2, p-Fox3a/Fox3a, p-PI3K/PI3K and p-Akt/Akt↓	Invigorating Yang, detoxifying and anti-cancer	Guo et al. (2022)
Yanggan Huayu granule	Broussonetia papyrifera (L.) L'Hér. ex Vent. (Chu Shi), rhizome of Curcuma aeruginosa Roxb. (E Zhu), and stem and leaf of Lycopus lucidus Turcz. ex Benth. (Ze Lan)	—	HepG2, SMMC-7721 and L02 cells	Mice	—	3 g/kg/d, 0.1 mL/10 g	14 d	5-FU (positive control)	Suppresses growth, migration and invasion of HCC	Cleaved-Caspase3 and Cleaved-PARP↑, p-AKT and BCL-2↓	Activates blood circulation and resolves blood stasis, soothes the liver and relieves pain	Shen et al. (2022)

## 9 Discussion and summary

The Wnt/β-catenin signaling pathway is increasingly recognized as critically involved in HCC pathogenesis, driving intensified development of targeted inhibitors. Current small-molecule inhibitors—including PKF115-854, CGP049090, NC043, and CWP232228—disrupt β-catenin/TCF4 complex formation. OMP-18R5 inhibits Wnt ligand binding to Frizzled receptors (Liu H. Y. et al., 2024). These pharmacologic agents typically engage single, precise targets to block Wnt/β-catenin signaling, though clinical validation remains pending. No Wnt/β-catenin-centric drugs are currently approved for HCC.In contrast, TCM exhibit polypharmacological actions, simultaneously targeting multiple nodes of the Wnt/β-catenin cascade: nuclear translocation blockade of β-catenin, activation of the β-catenin destruction complex, and suppression of Wnt ligands. This multi-target engagement confers unique potential for synergistic enhancement of conventional therapies. For instance, constituents like Astragalus polysaccharides may augment T-cell activity while alleviating immunosuppressive microenvironments, potentially potentiating ICIs efficacy.

Contemporary targeted therapies (e.g., sorafenib, lenvatinib) and ICIs universally confront primary or acquired resistance in HCC management. TCM may circumvent resistance through multifaceted modulation of relevant pathways, such as disrupting therapeutic resistance by modulating β-catenin/TCF-4 complex assembly, suppressing β-catenin nuclear translocation, and inhibiting EMT. Regarding ICI resistance, TCM may remodel the tumor immune microenvironment via regulating immunosuppressive cells (e.g., Tregs, MDSCs), enhancing antigen presentation, and suppressing non-PD-1/PD-L1 immune checkpoints, thereby restoring ICI responsiveness. Crucially, TCM leverages syndrome differentiation (辨证论治) as its core paradigm. HCC patients exhibit dynamic syndrome evolution (e.g., Liver Qi Stagnation with Spleen Deficiency, Dampness-Heat Accumulation, Liver-Kidney Yin Deficiency, Blood Stasis-Toxin Interaction) during modern therapies, which interact with treatment-induced adverse manifestations like heat-toxicity (热毒), damp-turbidity (湿浊), and qi-blood depletion (气血亏虚). Consequently, the most promising integrative strategy involves precision-guided formulation of Chinese herbal formulas based on individual syndrome trajectories, therapeutic phases, and toxicity profiles.

It is important to note that current studies on the mechanism of HCC inhibition by TCM are still mainly limited to the preclinical research stage. Most of the mechanism explorations reported in the existing literature are based on *in vitro* cellular experiments (e.g., HCC lines such as HepG2 and SMMC-7721) and animal models (e.g., xenograft tumor models), focusing on the regulation of the key signaling pathway of Wnt/β-catenin, as well as the inhibition of biological processes such as tumor cell proliferation, migration, and the induction of tumor cell apoptosis. The translation of these preclinical findings into viable clinical therapeutics confronts critical barriers: first, species-specific discrepancies in pathological contexts between experimental models and humans prevent animal systems from replicating the synergistic pharmacokinetics of multicomponent TCM. This limitation drives supraphysiological exposures of bioactive constituents during high-dose, short-term interventions that far exceed clinically attainable concentrations (typically <2 mg/L).; Concurrently, neither tumor heterogeneity nor interpatient variability has been adequately addressed. Pharmacokinetically, key bioactive constituents (e.g., baicalin) exhibit oral bioavailability frequently below 10% due to intestinal enzymatic degradation and first-pass metabolism, substantially diminishing theoretical efficacy. Furthermore, quality control challenges arise from inconsistent botanical sourcing and non-standardized processing protocols, potentially introducing batch-to-batch variability that compromises therapeutic consistency. Standardization and quality control of TCM are essential for enhancing its identification and application in treating HCC. The multifaceted, multi-target, and multi-effect characteristics of TCM render chemical analysis insufficient for thoroughly assessing its quality and efficacy. Recent research has increasingly demonstrated that high specificity and sensitivity biomarkers can assess TCM’s quality, safety, and efficacy. Essential proteins in the Wnt/β-catenin signalling pathway, such as β-catenin, GSK-3β, Cyclin D1, and c-myc, are associated with HCC’s onset, progression, proliferation, migration, and apoptosis. They are anticipated to serve as possible indicators for HCC prognosis. Furthermore, enhancing the quality control of TCM for preventing and treating HCC facilitates the standardization of TCM.

Finally, the predominantly Chinese-led preclinical and clinical studies discussed herein introduce geographic concentration biases that may propagate publication bias (e.g., preferential reporting of positive results with potential underreporting of negative/null findings) and language bias (limited to Chinese/English literature), potentially compromising evidence comprehensiveness despite systematic searches across multiple databases (PubMed, Web of Science, CNKI)—thus necessitating cautious interpretation of conclusions’ generalizability. Furthermore, supporting animal models harbor inherent limitations: species-specific metabolic disparities prevent recapitulation of TCM’s holistic regulatory properties, while clinically irrelevant dosing regimens (e.g., high-dose short-term interventions) constrain extrapolation to human efficacy. Consequently, the core evidentiary bottleneck resides in the paucity of high-quality RCTs, with existing studies frequently compromised by inadequate sample sizes, deficient randomization/blinding, clinically irrelevant endpoints, and unsystematic adverse event reporting. Future research urgently requires multicenter large-sample RCTs adhering to CONSORT standards—incorporating prospective registration, rigorous allocation concealment, blinded assessment, patient-reported outcome metrics, and standardized botanical quality control—as only such methodologically rigorous clinical validation can elevate TCM’s evidence-based standing in modern medicine.

## Abbreviations

APC, Adenomatous polyposis coli; AXIN1, Axis inhibition protein 1; Bcl-2, B-cell lymphoma-2; CCA, Cholangiocarcinoma; CK 1α, Casein kinase 1α; CTNNB1, Catenin Beta 1; DCs, Dendritic Cells; DVL2, Dishevelled-2; EMT, Epithelial-Mesenchymal Transition; EpCAM, Epithelial cell adhesion molecule; FZD, Frizzled; GSK-3β, Glycogen Synthase Kinase-3β; HCC, Hepatocellular carcinoma; ICIs, Immune checkpoint inhibitors; LEF, Lymphoid enhancer factor; LRP, Low Density Lipoprotein Receptor-Related Protein; M2-TAM, M2 tumour-associated macrophages; TACE, Transcatheter arterial chemoembolization; TCF, T cell-specific transcription factor; TCM, Traditional Chinese medicine; Th, T helper; TME, Tumor microenvironment; VEGF, Vascular Endothelial Growth Factor.
